# Hydrophilic and Amphiphilic Macromolecules as Modulators of the Physical Stability and Bioavailability of Piribedil: A Study on Binary Mixtures and Micellar Systems

**DOI:** 10.1021/acs.molpharmaceut.5c00276

**Published:** 2025-06-30

**Authors:** Luiza Orszulak, Aldona Minecka, Roksana Bernat, Taoufik Lamrani, Karolina Jurkiewicz, Barbara Hachuła, Magdalena Tarnacka, Monika Geppert-Rybczyńska, Maciej Zubko, Marcela Staniszewska, Michał Smoleński, Justyna Dobosz, Grzegorz Garbacz, Kamil Kamiński, Ewa Kamińska

**Affiliations:** † Institute of Chemistry, Faculty of Science and Technology, 431562 University of Silesia in Katowice, Szkolna 9, 40-006 Katowice, Poland; ‡ Department of Pharmacognosy and Phytochemistry, Faculty of Pharmaceutical Sciences in Sosnowiec, Medical University of Silesia in Katowice, Jagiellonska 4, 41-200 Sosnowiec, Poland; § Institute of Materials Engineering, University of Silesia in Katowice, 75 Pulku Piechoty 1A, 41-500 Chorzow, Poland; ∥ Institute of Physics, Faculty of Science and Technology, University of Silesia in Katowice, 75 Pulku Piechoty 1A, 41-500 Chorzow, Poland; ⊥ Department of Physics, Faculty of Science, University of Hradec Králové, Rokitanského 62, 500 03 Hradec Králové, Czech Republic; # Physiolution Polska sp. z o.o., Skarbowcow 81/7, 53-025 Wroclaw, Poland

**Keywords:** Soluplus copolymer, polyvinylpyrrolidone, piribedil, amorphous solid dispersions, micellar systems, topology of polymers, drug delivery system, solubility, bioavailability

## Abstract

This study presents an innovative approach that utilizes polymers with different topologies and properties as potential matrices for the poorly water-soluble active pharmaceutical ingredient piribedil (PBD). We investigated amorphous solid dispersions (ASDs) as well as micellar systems composed of PBD and (*i*) the commercial amphiphilic copolymer Soluplus, (*ii*) self-synthesized hydrophilic linear PVP (*lin*PVP), and (*iii*) self-synthesized hydrophilic star-shaped PVP (*star*PVP). Differential scanning calorimetry, X-ray diffraction, Fourier-transform infrared, and broadband dielectric spectroscopy were applied to gain comprehensive insights into the thermal and structural properties, intermolecular interactions, global molecular dynamics, and recrystallization of the API from the amorphous PBD–polymer ASDs. The primary objective was to evaluate the impact of the type and topology of macromolecules, as well as the composition of binary formulations, on the physical stability of PBD in the amorphous form, phase transition temperatures, the API’s recrystallization rate, and ultimately, the release of drug in the prepared ASDs and micelles. Most importantly, our research led to the discovery of new polymorphic form (II) of PBD that has not been previously described in the scientific literature. We also revealed that ASDs containing hydrophilic PVP polymers exhibit the best performance in stabilizing the amorphous form of the API, with the *star*PVP systems showing the highest stabilization effect. In contrast, for micellar systems, Soluplus turned out to be the most suitable candidate in terms of forming the self-assembles of the lowest size distribution among all systems. The long-term stability of the amorphous drug in PBD–Soluplus micelles was higher compared to PBD–*star*PVP ASD. Moreover, an improvement in the bioavailability of the API contained in all tested formulations (binary and micellar systems) was observed, with PBD–*star*PVP micelles exhibiting the most desirable drug release profile within the polymer matrix, as well as the highest concentration of released drug. The obtained data highlight the crucial role of the type and topology/architecture of the polymer in the design of novel pharmaceutical formulations.

## Graphical abstract

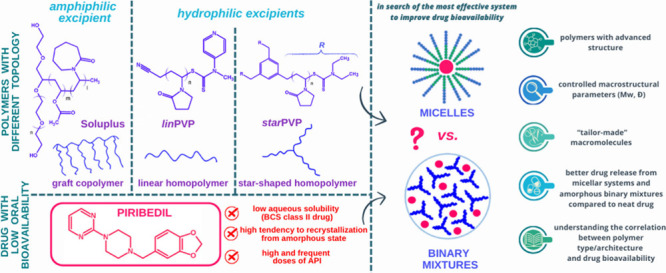

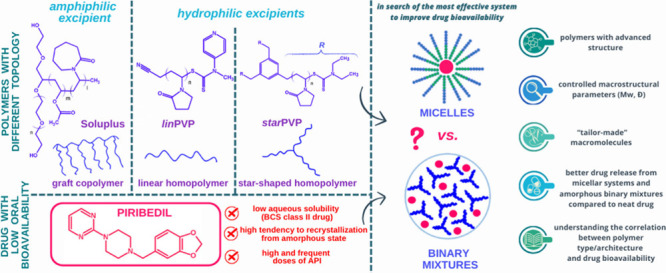

## 1 Introduction

The pharmaceutical industry is considered to be one of the fastest-growing sectors of the economy. However, despite this, it still struggles with a significant challenge—the poor water solubility (and consequently low bioavailability) of many active substances (APIs)/drugs available on the market, which results in their unsatisfactory therapeutic effect.^1–4^ Moreover, patients have often to take higher doses of pharmaceuticals, leading to undesirable side effects.^5^ One way to overcome these problems and improve the bioavailability of APIs is amorphization, i.e., the transformation of the initial crystalline substances into amorphous ones. The resulting material is characterized by a lack of long-range order compared to the crystalline form, which leads to improved solubility and bioavailability of APIs.^6,7^ However, amorphous substances are thermodynamically unstable, possess a high Gibbs free energy, and, as a result, show a high tendency to recrystallization, i.e., return to their energetically favorable crystalline form during storage or use of the products.^7,8^ To stabilize these systems, various excipients, EXCs (both low- and high-molecular-weight compounds) are widely applied. Among them, polymers are gaining popularity as innovative pharmaceutical additives.

It should be emphasized that polymers are considered as one of the most effective EXCs for stabilizing the labile amorphous form of APIs, due to numerous favorable properties.^9–12^ The key benefits of using them in pharmaceutical formulations include: *(i)* a high glass transition temperature (*T*_*g*_), which significantly increases *T_*g*_* of the entire drug-polymer system,^13^ *(ii)* reduction of the molecular mobility of the drug,^14,15^ *(iii)* an increase in the activation energy of API nucleation;^16,17^ *(iv)* the ability to synthesize “tailor-made” macromolecules adapted to specific types of drugs through various controlled polymerization methods, i.e., polymers with targeted molecular weights (*M_*w*_*) and low dispersity (*Đ*);^18^ *(v)* the possibility of modifying polymer chain ends to build subsequent polymer blocks and produce (co)­polymers, thereby fine-tuning macromolecular properties to suit specific applications.^19^ Given these unique features, polymers can significantly influence the dissolution, distribution, and transport of drugs within the human body. However, it is crucial to ensure that the polymer matrix is carefully selected for the specific API, the expected/desired properties, and also the used drug delivery system (DDS).

Among advanced DDSs, micellar systems and amorphous binary mixtures (BMs), also known as amorphous solid dispersions (ASDs), stand out as promising approaches for targeted therapy and controlled release.^20^ However, as mentioned earlier, for these new formulations to work effectively, the polymer matrix must be carefully selected for the specific DDS.^21^ Micellar DDSs primarily utilize amphiphilic polymers, which contain both hydrophilic and hydrophobic segments. Such a structure enables them to self-assemble in aqueous environments, forming micelles with cores capable of solubilizing hydrophobic APIs.^22^ These systems offer several advantages, such as enhanced drug stability, controlled release, and the ability to modify the micelle surface for tissue specificity. As a result, they are widely applied in formulations of APIs with low water solubility and targeted DDSs, including cancer therapies and vaccines.^23,24^ On the other hand, in amorphous BMs/ASDs, mostly hydrophilic polymers, due to their strong affinity to water, are primarily used to enhance the drug bioavailability by improving wettability, solubility, and dissolution rate. These systems may reduce or completely damp the crystalline order and stabilize disordered APIs, preventing their recrystallization and maintaining higher concentrations in solution.^12,25^ Among the well-known amphiphilic polymers, a copolymer Soluplus, deserves attention. There are increasingly frequent reports indicating its significant ability to enhance the solubility of hydrophobic APIs.^26–28^ Consequently, it has been proposed as a carrier for oral drug administration,^29,30^ ocular,^31,32^ and topical applications,^33,34^ as well as intravenous injections in cancer treatment.^27,35^ Due to amphiphilic properties, it can self-assemble into micelles with a hydrophilic outer shell and a hydrophobic core that entraps the hydrophobic drug, thereby facilitating its dissolution.^22^ Applying Soluplus in micellar DDSs with chosen APIs has been reported in several papers.^36–38^ There are also works that describe the impact of this polymer on the physical stability of amorphous APIs prepared by various methods,^39,40^ and even the liquid crystalline order of some pharmaceuticals, e.g., itraconazole.^41^ In turn, among hydrophilic macromolecules, one can mention polyvinylpyrrolidone (PVP), which is frequently used in various pharmaceutical formulations due to several exceptional properties (high *T_*g*_*, excellent water solubility, biocompatibility, nontoxicity, chemical stability, good adhesion, and emulsifying properties).^42,43^ It acts as an effective stabilizer for many amorphous APIs by reducing their molecular mobility through e.g., enhanced intermolecular interactions.^43,44^ Importantly, both approaches—micellar formulations and amorphous BMs, with the appropriate selection of polymer carriers—form the foundation of modern, advanced therapeutic systems, which not only enhance treatment efficacy but also minimize side effects, opening new possibilities in the design of effective medications.

It is important to highlight that, despite ongoing investigations into new polymeric-based pharmaceutical formulations, scientists still predominantly focus on applying various polymer matrices without delving into more complex aspects, such as polymer topology (linear and branched). However, it is well-known that macromolecules with the same chemical composition but differing in architecture/structure can exhibit distinct properties (e.g., various phase transition temperatures, hydrodynamic radius, degree of crystallinity, solubility, or number of functional groups at the chain ends).^45–48^ This suggests that such polymers might also cause varied effects on the bioavailability of active substances in drug-polymer formulations. Recognizing this overlooked scientific area, our research group has undertaken detailed research into the impact of macromolecular topology on the physicochemical and pharmacokinetic parameters of poorly bioavailable APIs. Preliminary studies on metronidazole-PVP systems demonstrated that the polymer topology influences drug-polymer interactions and miscibility (the branched polymer was miscible with the active substance in a wider range of concentrations compared to the linear macromolecules).^49^ Additionally, we revealed that the dispersity of the polymer is crucial for stabilizing amorphous forms of APIs.^49–52^ For instance, investigations on ASDs of the rapidly crystallizing drug—naproxen—and PVPs of varying topologies showed that macromolecules with tightly controlled parameters (targeted *M_w_*, and low *Đ*) effectively suppress the recrystallization of API from the amorphous form. In contrast, a commercially available PVP with high *Đ* (containing both high- and low-molecular-weight fractions) was the weakest inhibitor of the recrystallization process.^52^ Moreover, research on ASDs based on the extremely poorly water-soluble drug itraconazole demonstrated a significant improvement in API solubility (up to 20-fold) when dispersed in a star-shaped polymer matrix compared to a linear one.^51^ This clearly indicates that strict control over macromolecular parameters (such as *M_w_*, *Đ*) and architecture is crucial for designing advanced API-polymer formulations.

Inspired by previous intriguing results and aiming to further explore this fascinating scientific area, we developed new ASDs and micellar DDSs based on piribedil (PBD) – a poorly water-soluble and rapidly recrystallizing drug – and polymers with various architectures. As matrices, a commercially available amphiphilic graft copolymer known as Soluplus, composed of three distinct polymer blocks (polyvinyl caprolactam–polyvinyl acetate–polyethylene glycol, PCL–PVAc–PEG), as well as innovative, self-synthesized PVP matrices with linear (*lin*PVP) and three-arm star-shaped (*star*PVP) topologies, were selected. Applying PBD and the polymers described above, we created amorphous BMs and micellar systems in various API to EXC weight ratios, which were subsequently investigated using various experimental techniques. It should be clearly emphasized that just in this paper, using macromolecules of very similar molecular weight and different composition and topology, we have touched on all key aspects related to *(i)* the character of the polymer matrix (amphiphilic vs. hydrophilic), *(ii)* macromolecular topology (linear vs. branched), and *(iii)* the type of DDS (micelles vs. binary mixtures) to precisely determine which factors are important for improving the physical stability of API, drug release and consequently the bioavailability of the examined API. By closely following the results presented in this work, important conclusions can be drawn regarding the deliberate design of new drug-polymer pharmaceutical systems.

## 2 Materials and Methods

### 2.1 Materials

1-vinyl-2-pyrrolidone (VP, > 99%, *Sigma-Aldrich*) was passed through an alumina column before use to remove the inhibitor. 2,2′-Azobis­(2-methylpropionitrile) solution (AIBN, 0.2 M in toluene, *Sigma-Aldrich*), cyanomethyl methyl­(4-pyridyl) carbamodithioate (CTA1, 98%, *Sigma-Aldrich*), 1,3,5-tris­(bromomethyl)­benzene (97%, *Sigma-Aldrich*), sodium diethyldithiocarbamate trihydrate (*Sigma-Aldrich*), diethyl ether (pure for analysis, Chempur), methanol (99.85%, *PureLand*), dichloromethane (DCM, 99%, *Honeywell*), chloroform-d (99.8% D, contains 0.03% *v/v* TMS, *Sigma-Aldrich*), Soluplus (*M_w_* ∼118 000 g/mol, *Đ* = 2.05, *BASF*), crystalline PBD (IUPAC name 2-[4-(benzo­[1,3]­dioxol-5-ylmethyl)­piperazin-1-yl]­pyrimidine, 98%, *Angene*) were used as received. Acetonitrile for HPLC, ammonium acetate, sodium chloride, and anhydrous sodium dihydrogen phosphate were purchased from Th. Geyer Ingredients GmbH & Co. KG (Höxter-Stahle, Germany). Sodium hydroxide was purchased from VWR Chemicals (Leuven, Belgium). 3F Powder was obtained from the biorelevant.com LTD (London, United Kingdom). Pronoran (Les Laboratoires Servier, France) was purchased from the local pharmacy. Ultrapure water was self-produced from Hydrolab Ultra UV (Hydrolab Sp z o.o., Straszyn, Poland).

### 2.2 Methods

#### 2.2.1 Amorphous Binary Mixtures’ Preparation

Amorphous binary mixtures (BMs) composed of PBD and PVP polymers with different topologies, as well as commercial Soluplus polymer, were obtained by melt cooling method. They were prepared at different weight ratios of API to polymer, i.e., 90:10, 80:20, 70:30, and 60:40 *w/w*. To obtain a homogeneous mixture, appropriate amounts of crystalline PBD and polymer were weighed, carefully transferred to a metal plate, and preliminarily mixed using a spatula. Then, the plate with the API-polymer mixture was moved to a hot plate heated to a temperature of 403 K. After a while, PBD began to melt and the entire BM was stirred until complete dissolution of the macromolecule in the API. After determining a homogeneous system, each sample was vitrified by rapidly transferring it to a precooled copper plate.

#### 2.2.2 Drug Loading and Micelle Preparation

PVP or Soluplus and PBD were dissolved in chloroform CHCl_3_ with the following API-polymer weight ratios: 1:1 and 1:2. Solutions were added dropwise into deionized water and stirred overnight to evaporate the organic solvent. An excess of nonencapsulated drug was removed via filtration (using medium-graded filters with a pore size of 8–12 μm). In the final step, aqueous solutions were lyophilized (freeze-dried). More precisely, the obtained filtrate was frozen in liquid nitrogen for 10 min, then placed in a freeze-dryer (Labconco FreeZone 4.5 L) and lyophilized at 189 K and 0.1 mbar for 48 h.

#### 2.2.3 Thermogravimetric Analysis (TGA)

The degradation of the drug (PBD) as well as the pure excipients (Soluplus, *lin*PVP, and *star*PVP) was investigated using a Mettler TG 50 thermogravimetric analyzer coupled with a Mettler MT5 balance (Mettler Toledo, Switzerland). The powders were placed in aluminum pans and heated in a furnace under a nitrogen flow (30 mL/min) at a heating rate of 10 K/min, from room temperature up to *T* = 873 K. Degradation temperatures of the samples were determined based on the percentage of mass loss.

#### 2.2.4 Differential Scanning Calorimetry (DSC)

Preliminary calorimetric measurements of BMs containing PBD and various polymers (i.e., Soluplus, *lin*PVP, and *star*PVP) with different weights ratios (90:10, 80:20, 70:30, 60:40 *w/w*), as well as neat PBD were performed using a Mettler-Toledo DSC system, which is equipped with a liquid nitrogen cooling accessory and an HSS8 ceramic sensor. Temperature and enthalpy were calibrated using indium and zinc standards. Samples were placed in aluminum crucibles (40 μL). The examined PBD-Soluplus 90:10, 80:20, 70:30, and 60:40 *w/w* binary mixtures, as well as neat API were heated from 293 to 393 K, then cooled to 222 K, and reheated to 393 K. An analogous procedure (heating–cooling–heating) was applied in the case of PBD–PVPs 90:10, 80:20, 70:30, and 60:40 *w/w* binary mixtures, but within a different temperature range (they were heated in the *T*-range of 293–381 K, then cooled to 250 K, and reheated to 381 K). In turn, neat polymers (Soluplus, *lin*PVP, and *star*PVP) were heated from 293 to 483 K, next cooled to 230 K, and heated again to 425 K (Soluplus) or 483 K (PVPs). Measurements were performed at a constant heating/cooling rate (ϕ) of 10 K/min. In turn, nonisothermal studies at a ϕ from 2 to 20 K/min were carried out over a temperature range of 220 to 410 K. Additionally, the amorphous PBD-Soluplus and PBD-*lin*PVP binary mixtures (90:10, 80:20, 70:30, 60:40 *w/w*), as well as the molten API, were left to recrystallize at room temperature, after which each sample was scanned using a slow heating rate of 2 K/min within the temperature range of 298 to 413 K. The data collected in this manner were used to confirm the miscibility of the systems. For one representative sample, PBD-*lin*PVP 60:40 *w/w*, calorimetric measurements were performed at a standard heating rate (ϕ = 10 K/min) over three thermal cycles (heating from 298 to 473 K, cooling to 223 K, and reheating to 473 K). Furthermore, the PBD-*lin*PVP 80:20 *w/w* formulation was subjected to calorimetric analysis both before and after the release process, with heating conducted from 298 to 403 K at a standard rate (10 K/min). Each measurement at a given ϕ was repeated 2 times. For each experiment, a new sample was prepared. It should be mentioned that all described calorimetric measurements for the PBD–PVPs systems were performed immediately after sample preparation to avoid water absorption by the hygroscopic PVP.

The values of the calorimetric glass transition temperature (*T_g_*) for all samples were determined as the midpoint of the heat capacity increment. In turn, the crystallization and melting temperatures (*T*_*c*_ and *T*_*m*_) were obtained from the maximum of the exothermic and endothermic peaks in the thermograms, respectively.

#### 2.2.5 X-ray Diffraction (XRD)

XRD patterns of neat PBD, polymers, their BMs, and micellar systems were collected using a D/Max Rapid II diffractometer (Rigaku, Tokyo, Japan) equipped with a rotating Ag anode X-ray tube powered by 12 kW, a graphite (002) monochromator, and a two-dimensional curved image-plate detector. The powdered samples were probed in borosilicate glass capillaries of 1.5 mm diameter. The size of the collimated incident X-ray beam on the probed sample was 0.3 mm, and the wavelength was 0.5608 Å (Ag *K*_α_ line). The background from empty capillary was also measured and subtracted from the patterns collected for samples. All measurements were performed at a temperature of 293 K. Before XRD measurements, each sample was vacuum-dried in a desiccator for a minimum of 1 h to eliminate any moisture.

#### 2.2.6 Fourier-Transform Infrared (FT-IR) Spectroscopy

FTIR spectra were measured on the Nicolet iS50 spectrometer (Thermo Fisher Scientific, Massachusetts, USA) in the ATR (attenuated total reflectance) mode in the range of 4000–400 cm^–1^ at 293 K. The data were recorded at a spectral resolution of 4 cm^–1^, taking 16 scans. The high-temperature FTIR spectrum of PBD (at *T* = 393 K) was measured using a GladiATR accessory (Pike Technologies) coupled with an FTIR spectrometer in the range 4000–400 cm^–1^ (32 scans; spectral resolution of 4 cm^–1^). All examined binary mixtures were measured immediately after preparation to avoid water absorption by hygroscopic PVP polymers.

#### 2.2.7 Broadband Dielectric Spectroscopy (BDS)

Complex dielectric permittivity measurements (ε* (ω) = *ε* ^′^(ω)-*iε* ^″^ (ω)) of binary mixtures composed of PBD and various polymers (90:10 and 80:20 *w/w*) were performed using the Novocontrol Alpha dielectric spectrometer (Novocontrol Technologies GmbH & Co. KG, Hundsangen, Germany), with temperature control provided by a Quatro system, employing a nitrogen gas cryostat with a stability better than 0.1 K. The data were collected over a frequency range from 10^–1^ to 10^6^ Hz. The sample was placed between two stainless steel electrodes of a capacitor (diameter: 15 mm, gap: 0.15 mm) and mounted on a cryostat.

Molecular dynamics studies of PBD-Soluplus BMs were conducted within the *T*-range of 228–397 K. In turn, PBD-*lin*PVP and PBD-*star*PVP systems were measured in the *T*-range of 173–353 K.

Crystallization kinetics studies (neat PBD and PBD-polymer 90:10 and 80:20 *w*/*w* systems) were performed at a frequency of 10^4^ Hz, which corresponded to different temperatures depending on the system (please see Table 1 in the main manuscript). Each sample, after preparation, was heated to *T* = 373 K (above the melting point of the API), subsequently cooled well below the glass transition temperature (approximately *T* = 173 K), and then heated to the planned crystallization temperature. Spectra were collected until complete crystallization occurred.

**Table 1:** Parameters of the Avrami Formula Used to Describe the Kinetics of Isothermal Crystallization Monitored Using BDS for Neat PBD and PBD-Polymer Systems

***No.***	***Sample/System***	***Weight ratio API-polymer***	***T***_***c***_ ***[K]***	***k*** ***[s** ^**–1**^ **]***	***n*** ***-value***	***t*** **_*1/2*_** ***[s]***
1.	neat PBD	–	288	1.81 × 10^–4^	2.55	4 800
2.	PBD-Soluplus	90:10	328	2.57 × 10^–4^	3.00	3 400
3.	PBD-*lin*PVP		333	2.66 × 10^–4^	2.92	3 400
4.	PBD-*star*PVP		333	1.79 × 10^–4^	3.67	5100
5.	PBD-Soluplus	80:20	333	1.56 × 10^–4^	1.85	5 300
6.	PBD-*lin*PVP		345	6.37 × 10^–5^	2.74	13 800
7.	PBD-*star*PVP		341	5.21 × 10^–5^	3.85	17 700

#### 2.2.8 Critical Micelle Concentration (CMC)

The surface tension (γ) at 25 °C ± 0.1 °C was measured by the pendant drop technique with a Krüss DSA 100 tensiometer (using Advanced software). There is a direct method, in which γ is determined by fitting the Young–Laplace equation (eq 1) to the drop’s contour.

1 $$\Delta P=P_{\mathrm{int}}-P_{ext}=\gamma \cdot \left(\frac{1}{R_{1}}+\frac{1}{R_{2}}\right)$$

where *P*_*int*_, *P*_*ext*_ are pressures inside and outside of curved liquid surface/interface, respectively, γ is the surface tension, and *R*_1_ and *R*_2_ are the main radii of curvature.

The drop’s size and shape are related to the liquid’s surface tension when the drop is hanging freely from the tip of the needle (in equilibrium with the cohesive and gravitational forces). The resolution and the accuracy of the surface tension measurements declared by the manufacturer are 0.01 and 0.3 mN/m, respectively. The critical micelle concentration (CMC) of the surfactants in aqueous solution can be calculated from the analysis of the concentration (c) dependence of γ (as γ (c) or γ (logc)). It is determined as a cross-point of two straight lines representing lower and higher concentration dependences of γ for the surfactant (before and after CMC). It is well-known that for higher concentrations, after CMC, the surface tension changes only slightly since all aggregation processes occurring in the bulk solutions have a minimal influence on the surface.

#### 2.2.9 UV–Vis Spectrophotometry

All measurements were performed using Agilent Technologies Cary 60 UV–Vis spectrophotometer and Cary WinUV software for data processing. PBD solutions in ethanol of different concentration were measured to determine calibration curve (see **Figures S16 and S17** in the Supporting Information, SI).

Drug loading efficiency (DLE) and drug loading content (DLC) were determined for the prepared micellar systems dissolved in ethanol and calculated from the following eqs (eq 2) and (eq 3):

2 $$DLE=\frac{m_{DM}}{m_{D}}\cdot 100\%$$

where *m*_*DM*_ is the amount of drug in micelles, while *m*_*D*_ is the amount of drug used for preparing micellar systems.

3 $$DLC=\frac{m_{DM}}{m_{DLM}}\cdot 100\%$$

where *m*_*DLM*_ is the amount of drug-loaded micelles used for measurement.

For drug release studies, API-polymer micellar systems (30 mg) were dissolved in 1 mL of the Fasted State Simulated Intestinal Fluid (FaSSIF) solution, prepared before use according to the procedure described in the HPLC section. The solution was introduced into a dialysis cellulose membrane bag (MWCO 3.5 kDa), which was placed into a glass vial with 30 mL of FaSSIF solution and stirred at 37 °C in an oil bath. The dialysis was carried out for 24 h. The solution samples (100 μL) were taken from the release medium at appropriate time intervals (5, 15, 30, 60, 120, 180, 1200, and 1460 min) and dissolved in ethanol (1 mL) to determine the concentration of released drug by UV–Vis spectroscopy. Due to different DLC in each micellar system, the results were calculated referring to the equivalent of API used in HPLC studies to enable comparison of the drug release profiles performed utilizing these two methods (UV–Vis spectroscopy and HPLC).

#### 2.2.10 Dynamic Light Scattering (DLS)

Hydrodynamic diameters (*d_h_*) and zeta potentials (*ZP*) of polymer particles were measured on Malvern Zetasizer Nano-ZS (4 mW Hesingle bondNe ion laser, λ = 633 nm) for samples in deionized water (1 mg/mL) at 25 °C ± 0.1 °C.

#### 2.2.11 Transmission Electron Microscopy (TEM)

Microstructure analysis was carried out using JEOL JEM-3010 high-resolution transmission electron microscope (TEM, JEOL Ltd., Tokyo, Japan) with 300 kV acceleration voltage, equipped with a Gatan 2k × 2k Orius 833 SC200D CCD camera (Gatan Inc., Pleasanton, CA, USA). The studied micelles were suspended in isopropanol and deposited on a Cu grid with an amorphous carbon film standardized for TEM observations. Micelles' size distribution was characterized based on the image analysis performed using free-of-charge, open ImageJ software (1.54k).

#### 2.2.12 Preparation of FaSSIF Solution

Fasted State Simulated Intestinal Fluid (FaSSIF, biorelevant.com LTD, United Kingdom) was prepared using 3F Powder (FFF-02) according to the instructions of the manufacturer. Briefly, 0.42 g of sodium hydroxide, 3.438 g of anhydrous sodium dihydrogen phosphate, and 6.186 g of sodium chloride were weighed and dissolved in 900 mL of ultrapure water. The pH was adjusted to 6.5 with 1 M hydrochloric acid or 1 M sodium hydroxide and the volume was made up to 1000 mL. Then 2.24 g of 3F Powder was added. For equilibration, the solution was left at room temperature for 2 h.

#### 2.2.13 HPLC Method

The determination of PBD concentration in the micellar samples was determined using the RP-HPLC (Reverse Phase – High-Performance Liquid Chromatography) method adapted from Uppuluri et al.^53^ The analysis was performed on a Shimadzu LC-2050C system (Shimadzu U.S.A Manufacturing Inc., Canby, OR, USA) with a DAD detector, equipped with Phenomenex Kinetex EVO C18 (250 × 4.6 mm, 5 μm) chromatographic column. The mobile phase consisted of 10 mM ammonium acetate, pH 4.0, and acetonitrile (75:25) and the elution was isocratic. The flow rate was 1.5 mL/min. The column was thermostated at 40 °C. The retention time was 2.6 min, while the wavelength was 286 nm. The method was characterized by good linearity (*R* ^2^ = 0.9999).

#### 2.2.14 Determination of Saturation Solubility and Solubility Kinetics

Determination of the saturation concentration and solubility kinetics of PBD was performed in a small-scale dissolution system (Physiolution Poland). The device consisted of a magnetic stirrer with a precise thermostat, allowing the test to be carried out simultaneously in nine 25 mL glass vessels. An amount of sample equivalent to 25 mg of neat PBD was weighed into the vessel each time. In the Pronoran tablets test (50 mg of PBD, Les Laboratoires Servier, France) it was previously crushed in a mortar. Then 25 mL of FaSSIF preheated to 37 °C was added. The samples were stirred constantly, and the temperature was maintained at 37 °C.

Samples were withdrawn at the following time points: 5 min, 15 min, 30 min, 1, 2, 3, 4, 23, and 24 h. The sample was withdrawn through a 1 μm polyethylene cannula filter (ProSense B.V., Oosterhout, Netherlands) and then filtered into a centrifuge tube through a syringe filter (RC 0.2 μm, J.T. Baker). Subsequently, the sample was diluted with acetonitrile at a 1:1 volume ratio and analyzed by HPLC.

## 3 Results and Discussion

### 3.1 Results of Experimental Studies on ASDs Composed of PBD and Different Polymers

At the outset, it should be emphasized that herein we explore new-synthesized hydrophilic PVP matrices with various topologies (linear vs. branched) in different pharmaceutical formulations (binary systems and micelles) and compare them to a commercial amphiphilic copolymer (Soluplus) in the context of their impact on physical and pharmacokinetic properties of a hydrophobic drug, piribedil (PBD) used in the treatment of Parkinson’s disease. It is worth mentioning that the detailed procedures for obtaining PVPs with various topologies have been outlined in our previous publications.^49,51^ However, for the purpose of this work, we synthesized new linear PVP (*lin*PVP) and star-shaped PVP (*star*PVP) macromolecules with *M_w_* comparable to the commercial copolymer Soluplus, using the same synthetic procedures but modifying the reagent ratios, temperature, and reaction time (please see the SI). Such an approach allowed us to evaluate the influence of structure (linear vs. branched) and properties (hydrophilic vs. amphiphilic) of the polymer exclusively on the physical stability and the release (hence bioavailability) of PBD, eliminating the effect of EXC’s molecular weight on the behavior of the API. The chemical structures of PBD and the applied polymers, along with their macromolecular parameters, are presented in Figure 1.

**Figure 1:**
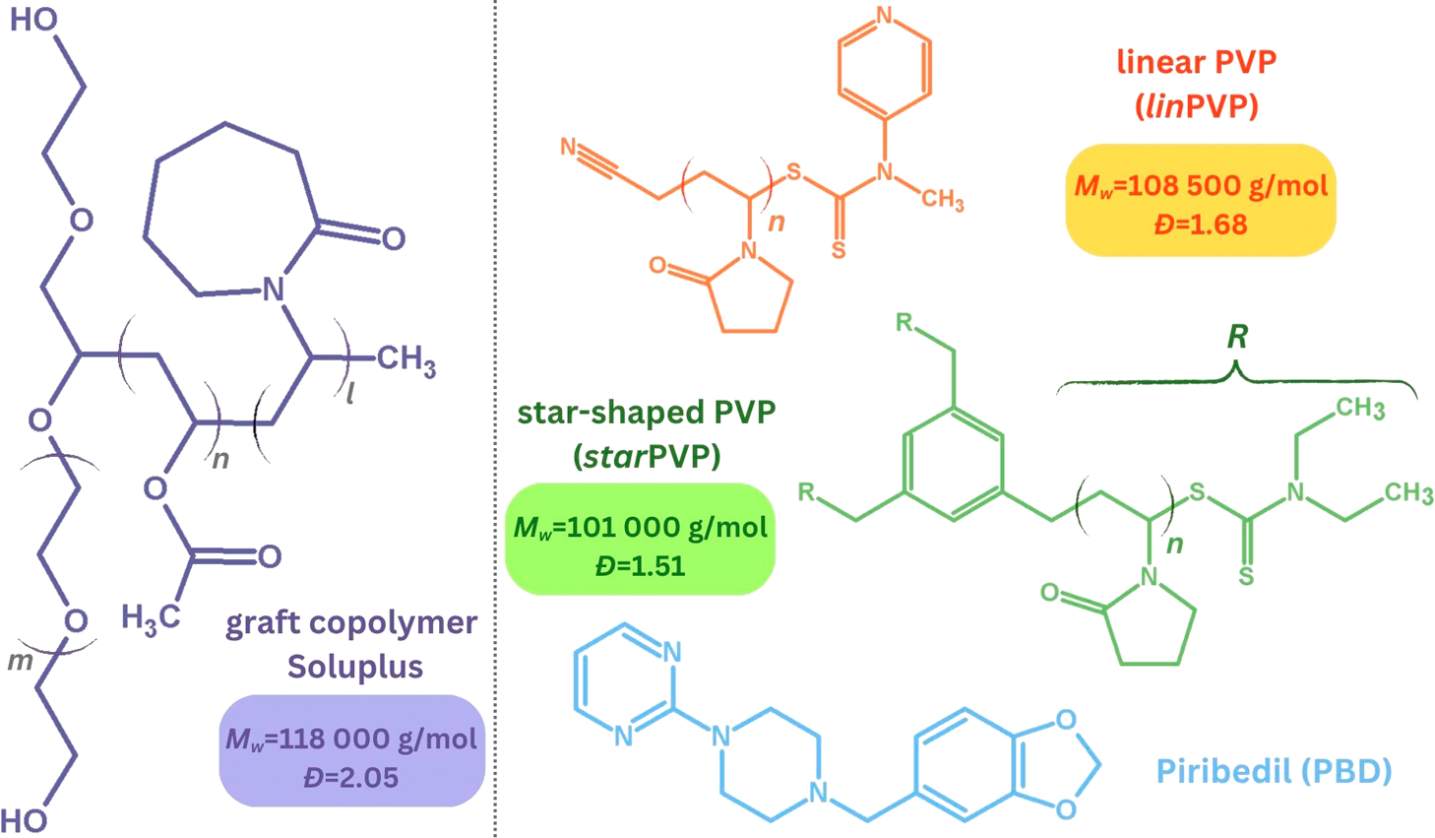
Chemical structures of PBD and different polymeric matrices (commercially available Soluplus and self-synthesized macromolecules – *lin*PVP and *star*PVP) along with their macrostructural parameters (*M_w_*, *Đ*).

Initially, before proceeding with the preparation of API–polymer mixtures, TGA analyses were performed for all pure substances to exclude the possibility of drug or polymer degradation during formulation using the high-temperature melt method (at *T* = 403 K). As clearly shown in **Figure S1** in the SI, the degradation temperatures of each compound significantly exceed 500 K, which clearly indicates that the melt-cooling method, described in detail in the Materials and Methods section, can be successfully applied for the preparation of binary formulations. Subsequently, for the self-synthesized polymeric matrices (*lin*PVP, *star*PVP), we conducted NMR measurements to confirm the chemical structure and purity of the obtained compounds (**Figures S2 and S3** in the SI). With the molecular structure of the synthesized macromolecules determined and individual degradation temperatures established, the preparation of binary mixtures was initiated. During multiple formulation attempts using the vitrification method, we found that PBD can mix freely with the excipients at a maximum weight ratio of 60:40 *w/w*. However, to verify this experimentally, NMR spectra were recorded for both physical mixtures and binary systems at the highest tested mass ratio (i.e., 60:40 *w/w*). As shown in **Figures S4** and **S5** in the SI, both physical mixtures and binary systems exhibit identical chemical shifts and comparable signal intensities, which confirms that the intended drug-to-polymer ratio was preserved during the preparation of binary mixtures. Next, DSC measurements were performed up to 473 K (i.e., above the *T_g_* of the neat polymer) for a selected representative PBD–*lin*PVP 60:40 *w/w* mixture. As seen on the representative thermogram (**Figure S6** in the SI), no signal corresponding to unmixed/free polymer is observed, suggesting the formation of a homogeneous binary mixture.

Nevertheless, to further confirm drug–polymer miscibility, a Flory–Huggins analysis was carried out. According to this approach, the interaction parameter (χ) can quantitatively describe miscibility from a thermodynamic perspective by using the melting point depression method.^54,55^ Typically, in miscible systems, due to the exothermic nature of mixing, a decrease in the melting point of the drug is expected, whereas the opposite trend indicates immiscibility. The determined χ values were negative, which indicates that the polymers used (Soluplus and PVPs) can be homogeneously mixed with the drug up to a 60:40 weight ratio. This analysis is presented and described in detail in the SI (**Figures S7** and **S8**). As a result, amorphous PBD–Soluplus and PBD–PVPs systems at 90:10, 80:20, 70:30, and 60:40 *w/w* were selected for further detailed analysis.

#### 3.1.1 DSC Data

At the beginning, we performed calorimetric measurements on the neat macromolecules (**Figure S9** in the SI) and API. As seen in Figure 2a, during the heating of crystalline PBD (ϕ = 10 K/min), a strong endothermic peak at *T* = 370 K corresponding to the melting of the substance, is visible in the thermogram (dark blue line). After cooling the molten sample (at the same ϕ = 10 K/min; navy blue line) and reheating it, four thermal events can be detected (light blue line). The first, occurring at lower *T*, might be attributed to the glass transition at *T_g_* = 260 K. Additionally, at higher temperatures, exothermic and endothermic processes, corresponding to the crystallization (at *T*_*c*_ = 327 K) and melting (at *T*_*m*(*I*)_ = 371 K, and *T*_*m*(*II*)_ = 364 K), respectively, are observed. The presence of the two melting peaks is an interesting observation that may indicate the formation of two polymorphic forms of PBD during the second heating run **(** Figure 2a, light blue line). To the best of our knowledge, only one polymorphic form of PBD (form I) with a melting point at *T*_*m*(*I*)_ = 370 K,^56^ has been reported in the literature so far. There are no mentions of the other polymorphs. This issue will be elaborated in the further part of this paper. Additionally, before proceeding with the calorimetric studies of the binary mixtures, it is important to highlight one crucial aspect. During the cooling of neat PBD from the molten state at a rate of ϕ = 10 K/min (Figure 2a, second scan, navy blue line), no signs of crystallization were observed. However, in the subsequent heating cycle (Figure 2a, third scan, light blue line), recrystallization of the API can be observed. This observation is important for correctly classifying PBD within the appropriate Glass-Forming Ability (GFA) group. The GFA classification system is based on the tendency of a drug to recrystallize during cooling and heating cycles.^57^ GFA class I refers to compounds that recrystallize during cooling of the melt at a rate of 20 K/min. Class II compounds do not recrystallize during cooling at 20 K/min but recrystallize during the subsequent heating cycle at 10 K/min. Finally, GFA class III compounds do not recrystallize during either cooling at 20 K/min or reheating at 10 K/min.^58^ The GFA classes (I, II, and III) represent poor, moderate, and good glass formers, respectively. Based on this classification, the studied drug should be assigned to GFA class II, indicating that PBD exhibits moderate stability of its amorphous form upon heating above its *T_g_*.

**Figure 2:**
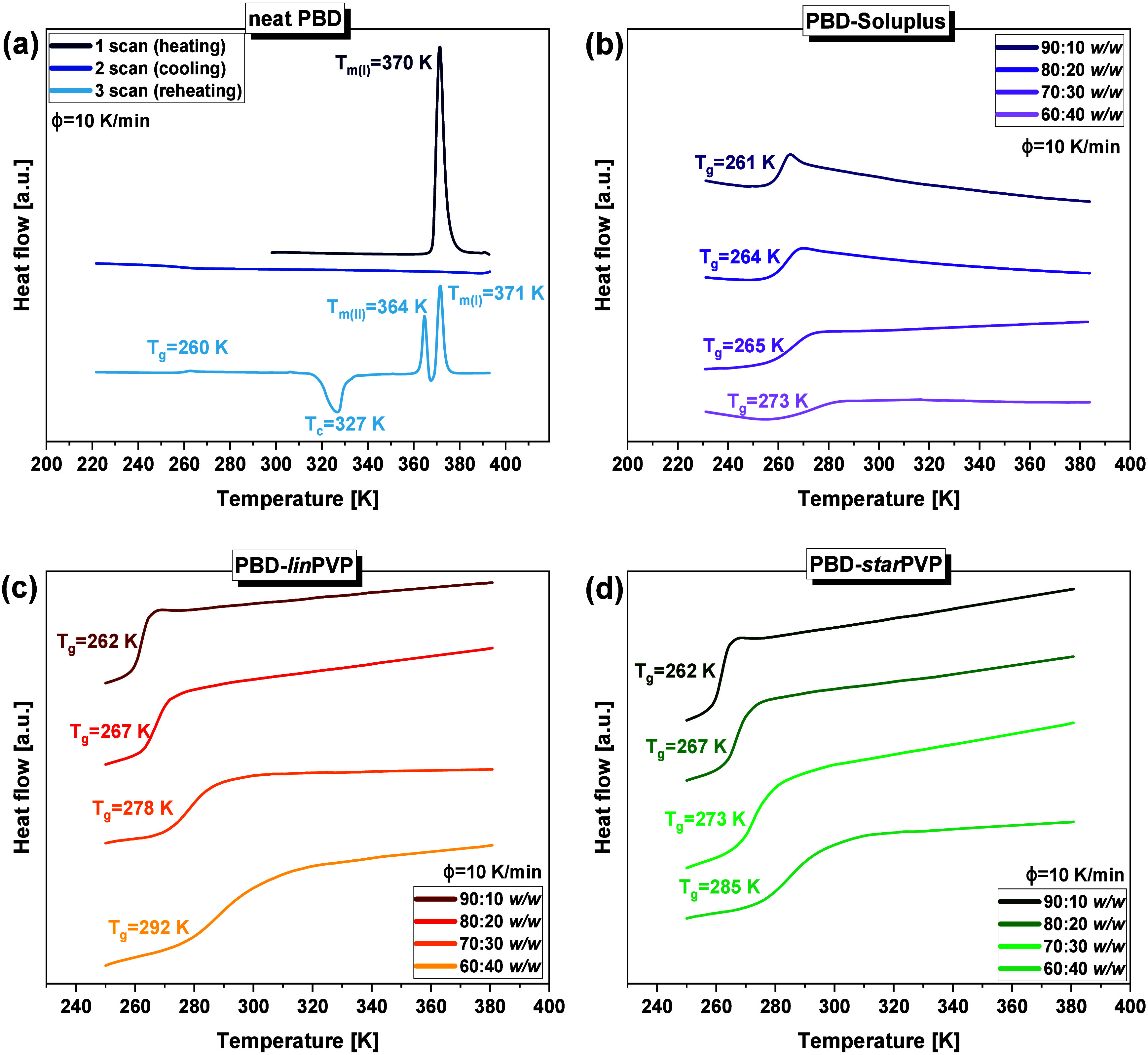
DSC thermograms (ϕ = 10 K/min) of **(a)** neat drug-PBD and binary mixtures with different weight ratios, **(b)** PBD-Soluplus, **(c)** PBD-*lin*PVP, and **(d)** PBD-*star*PVP (90:10, 80:20, 70:30, 60:40 *w/w*).

Intrigued by the above results, subsequent calorimetric measurements were carried out on PBD-polymer systems (also at the standard ϕ = 10 K/min) to check how the addition of EXCs affects the crystallization process of API and the formation of different polymorphs. As shown in Figure 2b–d, PBD in each mixture, even with a small amount of the excipient (10 wt %), could be vitrified regardless of the type of polymer matrix used. In every case, attempts to prepare ASDs were successful, and the DSC curves of these formulations showed only a single thermal event. Specifically, the thermograms of API-polymer systems reveal only the glass transition event without any signs of recrystallization, indicating their amorphous nature and the suppression of PBD crystallization. Values of *T_g_* determined for the individual BMs are presented in Figure 2 and additionally summarized in tabular form (please see **Table S1** in the SI).

They were also plotted as a function of the weight fraction of PBD in Figure 3. As expected, the lower *X*_*PBD*_ (and consequently, the greater *X*_*polymer*_ in the mixtures), the higher *T_g_* of the system. A similar scenario was reported in numerous works devoted to various API-EXC systems, both containing low-molecular-weight^59–62^ and high-molecular-weight^50–52,63,64^ additives. Therefore, the result obtained for the examined PBD-Soluplus and PBD-*lin*/*star*PVP BMs is not particularly surprising, but another aspect should be noted. Namely, the progressive addition of both PVP polymers (linear and branched) significantly influences the *T_g_*, while mixing PBD with Soluplus (10–30 wt % of the polymer) results in a minor variation in the *T_g_* (up to 4 K; see the purple squares in Figure 3). Only the larger amount of this macromolecule (40 wt %) causes a more pronounced increase in the glass transition temperature (up to 273 K), though still not as strong as in the case of the BMs with various PVPs. The observed effect is most likely related to the significant differences in the *T_g_* values of two PVPs and Soluplus polymers (see **Table S1** in the SI). At this point, it is worth highlighting an interesting observation – namely, despite the pronounced differences in the *T_g_* values of the pure polymers Soluplus and PVPs (approximately 100 K, see **Figure S9** in the SI), the binary mixtures (especially at the 90:10 *w/w* ratio) do not exhibit such striking discrepancies in *T_g_*. This phenomenon suggests a lack of specific intermolecular interactions between the polymers and the API, which was confirmed by infrared spectroscopy studies (description below). However, it should be noted that the absence of significant differences between the *T_g_* of BMs was mainly observed at low/moderate polymer concentrations (10 and 20 wt %). In contrast, as the polymer content increases, the *T_g_* values of individual ASDs begin to diverge more clearly, which corresponds well with the variations in *T_g_* observed for the pure excipients.

**Figure 3:**
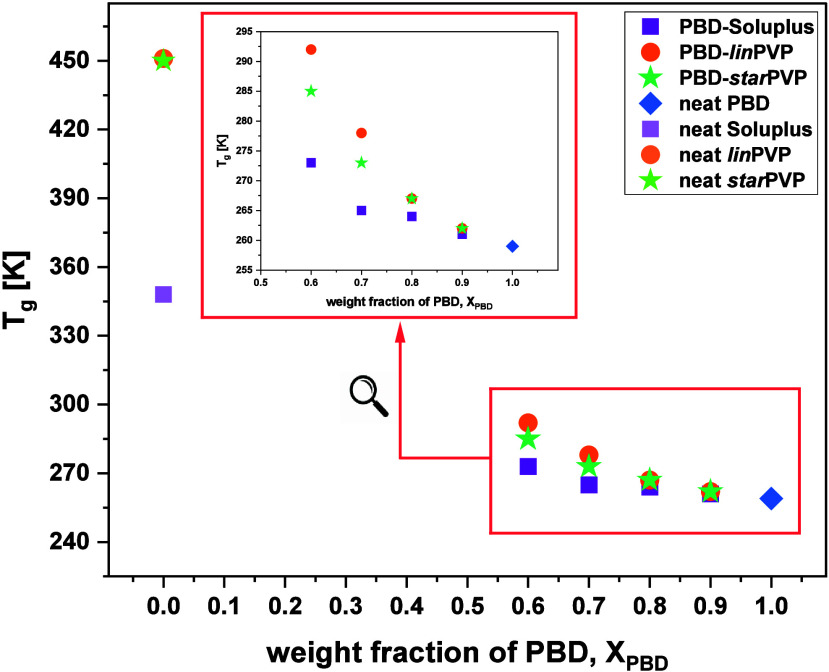
Dependency of calorimetric *T_g_* vs. X_PBD_ for the examined ASDs (ϕ = 10 K/min).

Taking into account the preliminary DSC measurements at a standard heating rate of 10 K/min, which demonstrated rather good physical stability and lack of crystallization of all tested BMs during heating, in the next step we conducted nonisothermal DSC studies at ϕ = 2, 4, 8, and 20 K/min for PBD and at ϕ = 2, 4, 6, and 8 K/min for the selected PBD-polymer BMs (Figure 4).

**Figure 4:**
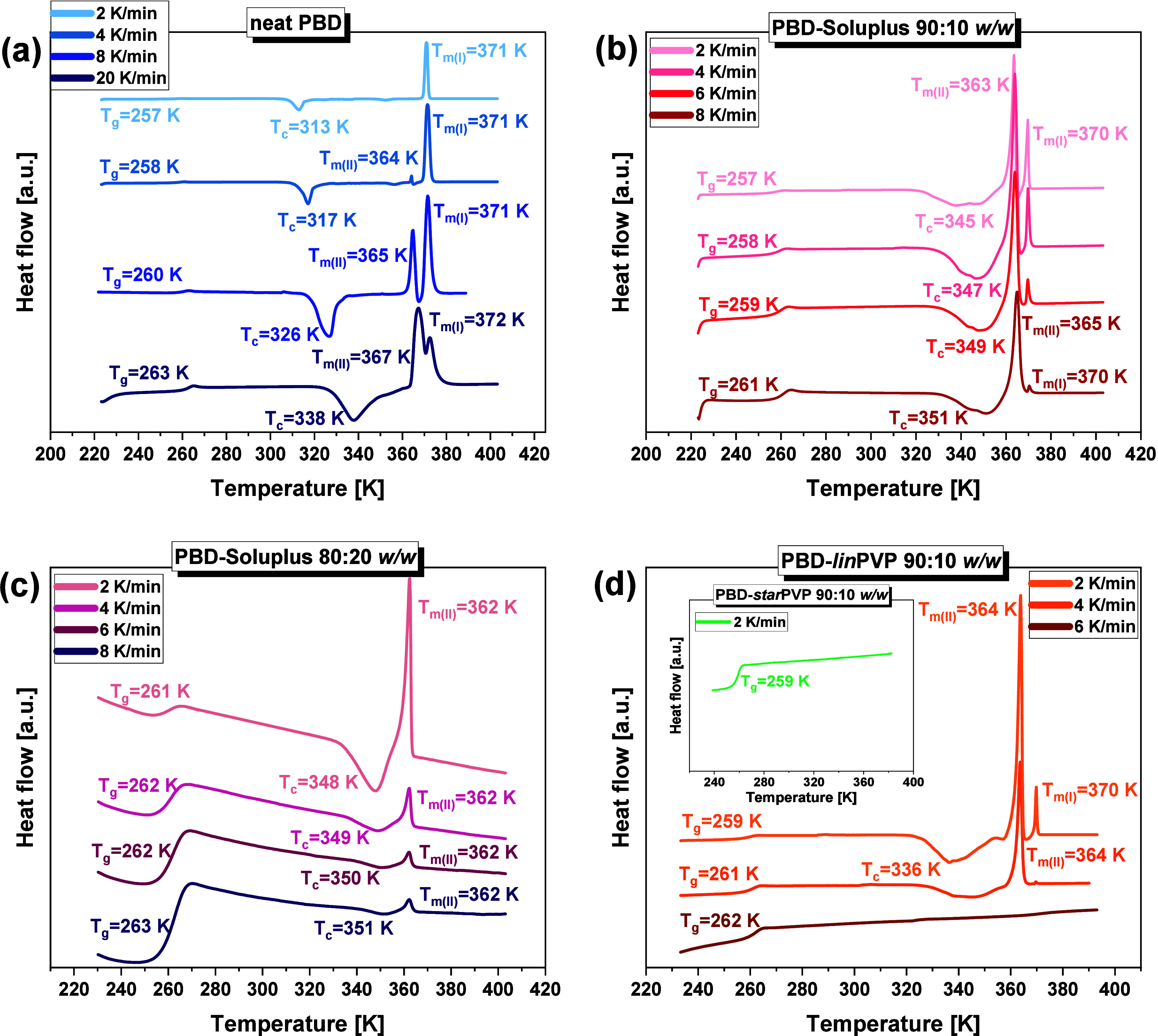
DSC thermograms obtained from nonisothermal measurements of **(a)** neat PBD, as well as different BMs: **(b)** PBD-Soluplus 90:10 *w/w*, **(c)** PBD-Soluplus 80:20 *w/w*, **(d)** PBD-*lin*PVP 90:10 *w/w* (in the inset, the thermogram of PBD-*star*PVP 90:10 *w/w* system collected at ϕ = 2 K/min is presented).

As shown in Figure 4a, the DSC curve of neat API reveals three/or four thermal events depending on the applied ϕ. As previously mentioned, the heat capacity jump at the lowest *T* corresponds to the glass transition (at *T_g_*). It is followed by a strong nonsymmetric exothermic signal attributed to the PBD crystallization (at *T*_*c*_), and an endothermic peak/or peaks related to the melting of API. In agreement with other reports on the nonisothermal studies,^65–67^ it can be observed that at higher ϕ, *T_g_* and *T*_*c*_ shift to higher values. Importantly, even at the highest ϕ (20 K/min), a strong exothermic peak (consisting of the two components) is well visible, which clearly suggests a great tendency of PBD to recrystallization. Moreover, at the slowest rate (ϕ = 2 K/min), only a single sharp and intense endothermic peak is noticeable at *T*_*m*(*I*)_ = 371 K. Conversely, as the ϕ increases (4, 8, and 20 K/min), an additional thermal event begins to appear (and becomes more pronounced in the thermogram) at a slightly lower *T* (*T*_*m*(*II*)_ = 364–367 K), Figure 4a. This leads to the conclusion that by modulating the heating rate of the neat PBD, it is relatively straightforward to alter the proportion of the two polymorphic forms (I and II) presented in the sample.

The outcomes of the nonisothermal calorimetric measurements for PBD-Soluplus, 90:10 and 80:20 *w/w* mixtures, as well as PBD–PVP 90:10 *w/w* systems are shown in panels **b–d** of Figure 4. As can be observed, PBD-Soluplus 90:10 *w/w* BM, similar to the neat PBD, exhibits four distinct thermal events (at *T_g_*, *T*_*c*_, *T*_*m*(*I*)_ and *T*_*m*(*II*)_). Interestingly, unlike the neat API, in this mixture, the recrystallization to the polymorphic form II with the lower melting temperature (*T*_*m*(*II*)_) is favored. Moreover, the amount of polymorph I with the higher *T*_*m*(*I*)_ gradually decreases with increasing ϕ. Another intriguing observation is that a higher Soluplus content in the mixture (20 wt %) results in recrystallization exclusively to the polymorphic form II of PBD with *T*_*m*(*II*)_ = 362 K, as is seen in Figure 4c. In turn, a *lin*PVP appears to be a better candidate for effective suppression of PBD recrystallization. Specifically, the thermogram of PBD-*lin*PVP 90:10 *w/w* BM exhibits a wide (double) exothermic peak assigned to the crystallization only at the slowest ϕ = 2 and 4 K/min, whereas at a slightly greater ϕ (6 K/min), only a glass transition event is observed, indicating a fully disordered nature of the material (Figure 4d). In the case of PBD-*star*PVP system, even just a 10 wt % of the polymer at the slowest ϕ (2 K/min) significantly suppresses the drug crystallization, as evidenced by the occurrence of only heat capacity jump at *T_g_* = 259 K in the DSC curve (see the inset in Figure 4d). It is worth adding that in PBD-*lin*PVP 90:10 *w/w* BM, the API recrystallized to both polymorphic forms: I and II with the predominance of the latter one. The content of both polymorphs is clearly reduced at ϕ = 4 K/min with respect to ϕ = 2 K/min. Note that the crystallization temperatures for the studied samples are summarized in **Table S2** in the SI. It should also be mentioned that the nonisothermal calorimetric data for the PBD-*lin*PVP and PBD-*star*PVP, 80:20 *w/w* BMs were not presented in Figure 4 due to the lack of recrystallization tendency even at the slowest ϕ (=2 K/min).

All the above observations lead to the conclusion that PVP matrices more effectively (in comparison to Soluplus) inhibit undesired recrystallization of the API from the binary mixtures. Furthermore, besides the type of polymer, its topology (linear and branched) plays a crucial role in controlling the crystallization of PBD. It is also worth stressing that, by selecting an appropriate polymer and adjusting the heating rate of the system, it is possible to obtain (upon recrystallization) one or two polymorphic forms of API of various content in the sample. This provides a unique opportunity to relatively easily modulate the stability of PBD depending on the specific needs.

Subsequently, based on the obtained nonisothermal DSC data, a Kissinger analysis was performed to determine the activation energy for the crystallization of the examined API (*E*_*cr*_). It should be added that in the case of API-PVP BMs, using such an approach (which is based on the variation of *T*_*c*_, i.e., the crystallization peak temperature, with ϕ) was not feasible due to the absence of crystallization phenomenon in the collected thermograms (this process was detected only for PBD-*lin*PVP, 90:10 *w*/*w* system at ϕ = 2 and 4 K/min - such data were insufficient to accurate analysis). Moreover, the PBD-*star*PVP BM at any weight ratio of both components and heating rate showed no signs of this process. Therefore, we applied the Kissinger method (eq 4)^68^ exclusively for PBD-Soluplus mixtures (see Figure 5)­

4 $$\ln\left(\frac{\phi }{{T_{c}}^{2}}\right)=C_{k}-\frac{E_{cr}}{RT_{c}}$$

**Figure 5:**
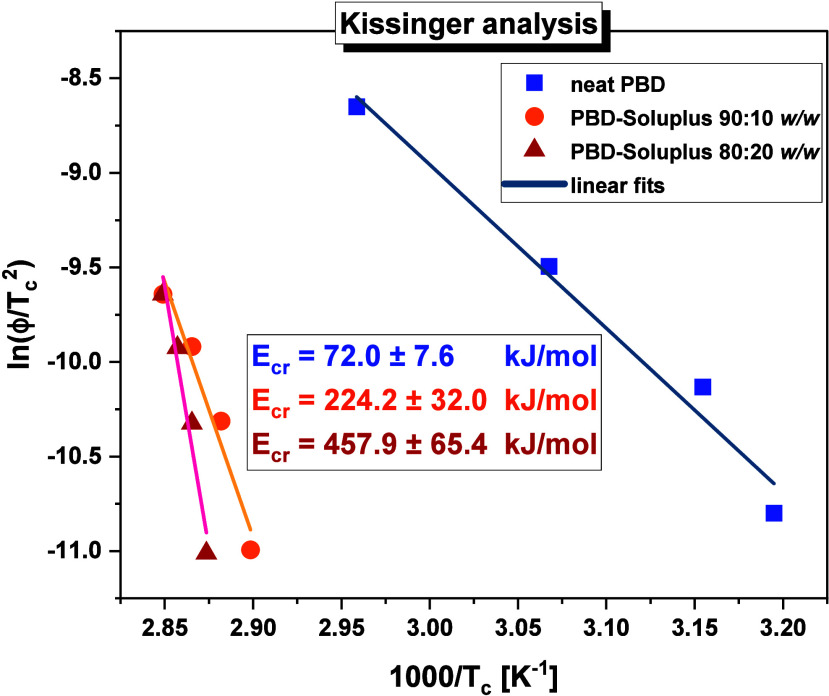
Kissinger plots for the exothermic crystallization peaks in neat PBD, as well as PBD-Soluplus 90:10 and 80:20 *w/w* systems (the solid lines represent linear fits).

where *C*_*k*_ is fitting parameter and *R* is gas constant.

As can be clearly observed in Figure 5, the activation barrier for the crystallization of PBD in BMs with Soluplus increases significantly compared to the neat API (*E*_*cr*_ = 72 kJ/mol). Even a 10 wt % of the polymer leads to a substantial increase in the *E*_*cr*_ value (up to 224 kJ/mol). For the PBD-Soluplus 80:20 *w/w* mixture, *E*_*cr*_ reaches up 458 kJ/mol. Such results indicate that this polymer (and its amount in the BM) has a significant impact on the activation barrier for the nonisothermal crystallization of PBD. At this point, it is important to emphasize that in the case of other API-polymer and API-oligosaccharide systems (with various amounts of EXC), such high *E*_*cr*_ values determined from the Kissinger analysis are rarely reported. The calculated activation energies for the drug crystallization are usually at a low/moderate level, e.g.,: for naproxen-various PVPs (*E*_*cr*_ = 68–77 kJ/mol),^52^ for metronidazole-PVPs (*E*_*cr*_ = 65–71 kJ/mol),^49^ and for metronidazole-cyclodextrins (*E*_*cr*_ = 51–68 kJ/mol).^61^ Higher values were determined for naproxen-oligosaccharides (*E*_*cr*_ = 64–109 kJ/mol),^62^ and flutamide-PVPs, 90:10 *w*/*w* ASDs (*E*_*cr*_ = 170–200 kJ/mol).^50^ Importantly, in the latter systems, in contrast to PBD-polymer ones, all macromolecules decreased slightly the activation barrier for flutamide crystallization in comparison to the neat API. To the best of our knowledge, only one study on the aspirin–poly­(vinyl alcohol-*co*-ethylene) revealed a great increase of *E*_*cr*_ (∼ 500 kJ/mol) compared to the neat drug (*E*_*cr*_ = 156 kJ/mol).^69^ The authors explained this finding as related to the inhibition of API crystal growth due to strong interactions between aspirin clusters and the polymer matrix.

In one of the further subsections, the outcomes of infrared studies, which were conducted to verify whether PBD-matrix interactions play a key role in inhibiting API crystallization, will be discussed.

#### 3.1.2 XRD Data

To specify the nature of the PBD phase structure in the studied binary systems with polymers as well as its stability and recrystallization behavior, we performed XRD studies. Figure 6 presents the comparison of the diffraction patterns for PBD-Soluplus **(panels a, d, g, j)**, PBD-*lin*PVP (**panels b, e, h, k**), and PBD-*star*PVP (panels **c, f, i, l**) mixtures at different weight ratios: 90:10, 80:20, 70:30, and 60:40 *w/w*, measured as fresh samples – just after preparation, and over time – over the course of days. All the experiments were carried out at room temperature (*T* = 293 K) and the samples were stored during this experiment in room conditions as well, in closed glass capillaries. The XRD patterns of neat PBD and polymers were also presented for reference. As one can see, the diffraction patterns of all BMs collected just after their preparation exhibit only a broad halo, confirming their amorphous character. However, in the case of the PBD-Soluplus system, the appearance of sharp Bragg peaks in the XRD data in the time scale of days is noted. PBD in ASD with the lowest concentration of Soluplus, 90:10 *w*/*w*, recrystallized after 1 day of storage, while for the higher Soluplus concentrations, there was a clear trend in the increase of the physical stability of the amorphous state with the higher fraction of the polymer. For the system with the highest loading of Soluplus (60:40 *w/w*), the recrystallization of PBD proceeded only after 2 weeks.

**Figure 6:**
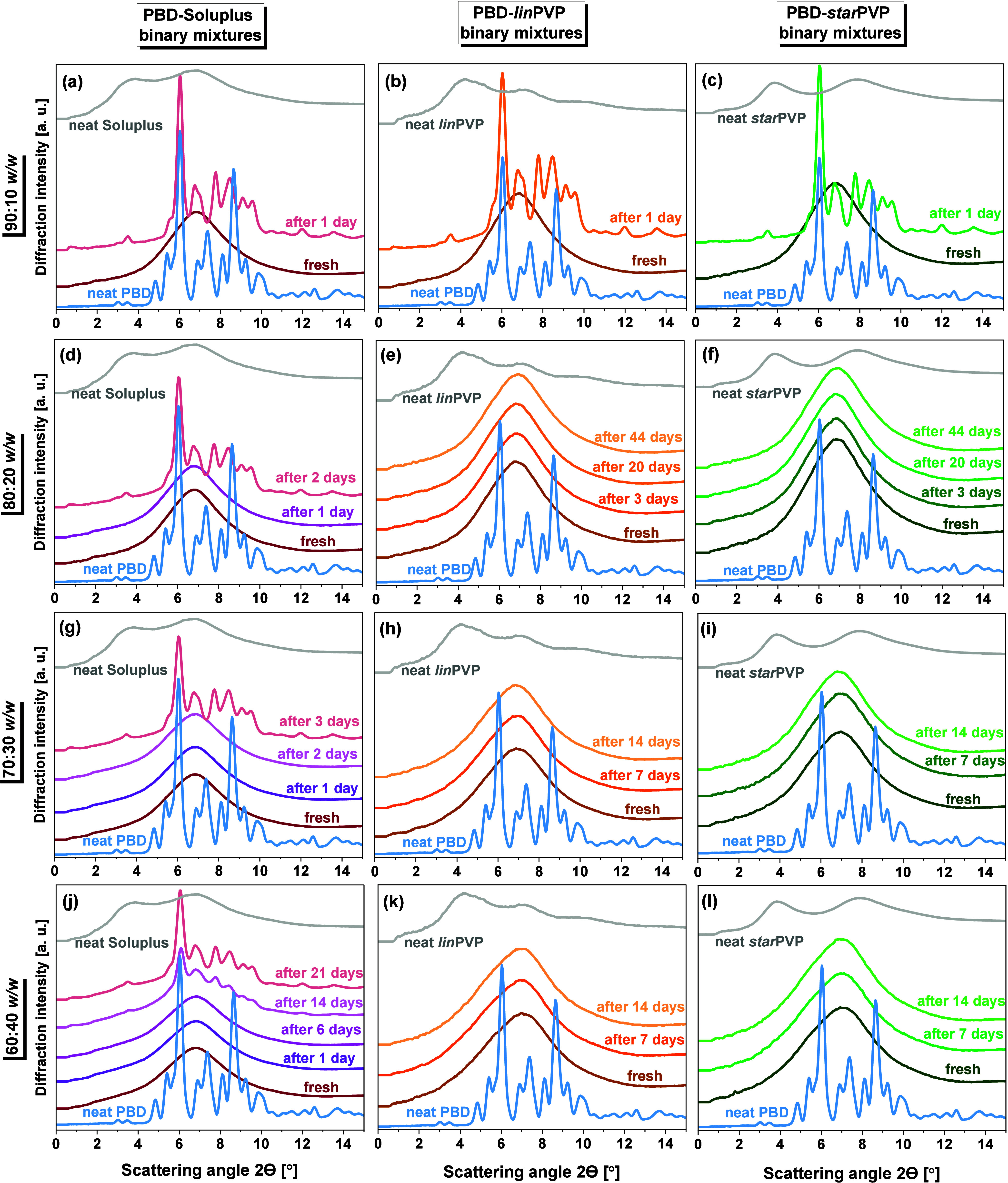
Temporal evolution of diffraction patterns for **(a, d, g, j)** PBD-Soluplus, **(b, e, h, k)** PBD-*lin*PVP and (**c, f, i, l**) PBD-*star*PVP mixtures at different weight ratios 90:10, 80:20, 70:30 and 60:40 *w/w*.

Regarding the polymorphic form of the PBD recrystallized from PBD-Soluplus ASDs, from the XRD patterns presented in panels **a, d, g, j** of Figure 6, one can see that the pattern of Bragg peaks for this recrystallized mixture is different from that reported for the neat PBD. For the 90:10 *w*/*w* system, the pattern for the recrystallized system was composed of Bragg peaks typical for polymorphic form I of the neat PBD as well as additional Bragg peaks (in the predominant fraction), which confirm the formation of another polymorphic phase (form II). Further calorimetric investigations on this BM recrystallized at room temperature confirmed this. However, a much different situation was found for the binary systems with the lower API content. XRD investigations supported by the further DSC studies on the recrystallized samples with the amount of Soluplus ≥ 20 wt %) indicated only one melting at *T*_*m*(*II*)_ = 360–361 K (see **Figure S10** in the SI). Therefore, we could conclude that the diffraction patterns collected for the BMs with the higher polymer content are strictly related to the sole presence of the polymorph II of PBD not described before in the literature.

To verify the stability of PBD in ASDs with PVP polymers (*lin*PVP and *star*PVP) in room conditions, their diffraction data measured at different time points were analyzed. As can be seen from **panels b** and **c** in Figure 6, the recrystallization of PBD in the systems with the lowest PVP content (90:10 *w/w*) was visible after 1 day of storage, regardless of the PVP architectures, similar to the behavior of the PBD in the system with Soluplus polymer of the same weight ratio. Moreover, the recrystallized systems were primarily composed of polymorphic form II, which agrees with the results of calorimetric investigations. In contrast, for ASDs with higher PVP concentrations (80:20, 70:30, and 60:40 *w*/*w*), no fingerprints of PBD recrystallization were observed up to 44 days. Thus, compared to the mixtures with Soluplus, the PBD–PVP BMs were much more physically stable.

#### 3.1.3 FTIR Data

The next step of our research was FTIR spectroscopy measurements. Their purpose, as previously mentioned, was to determine whether any specific interactions exist between the API and the polymers that could influence the different crystallization behaviors of PBD. The experiments were performed on the two representative BMs, namely PBD-Soluplus and PBD-*lin*PVP, at the highest possible weight ratio of the API and the polymer, 60:40 *w/w* (characterized by the greatest *T_g_*), for which potential intermolecular interactions should be most evident. At this point, it should also be explained that the PBD-*star*PVP 60:40 *w/w* system exhibits exactly the same spectral band pattern as the PBD-*lin*PVP 60:40 *w/w* mixture, which can be clearly observed in **Figure S11** in the SI. Therefore, in the following FTIR spectra figures, the comparisons mainly focus on the PBD-Soluplus and PBD-*lin*PVP 60:40 *w/w* BMs (as a representative of the PBD–PVP systems), ensuring good clarity and readability of the figures.

First, spectroscopic investigations were done on the neat substances (PBD, Soluplus, and *lin*PVP). In **Figure S12** in the SI, FTIR spectra of the individual components, with a detailed assignment of the spectral bands, are presented. To better identify the intermolecular interactions occurring between PBD molecules, the IR spectra of the API in the three different phases, i.e., the crystalline, glass (vitrified), and liquid (molten), were measured. These data, along with the spectra of API-*lin*PVP and API-Soluplus 60:40 *w/w* BMs in the supercooled liquid/or glassy states, were recorded at room temperature (*T* = 293 K) in the wavenumber regions of 3800–2400 and 1800–400 cm^–1^, see Figures 7 and 8.

**Figure 7:**
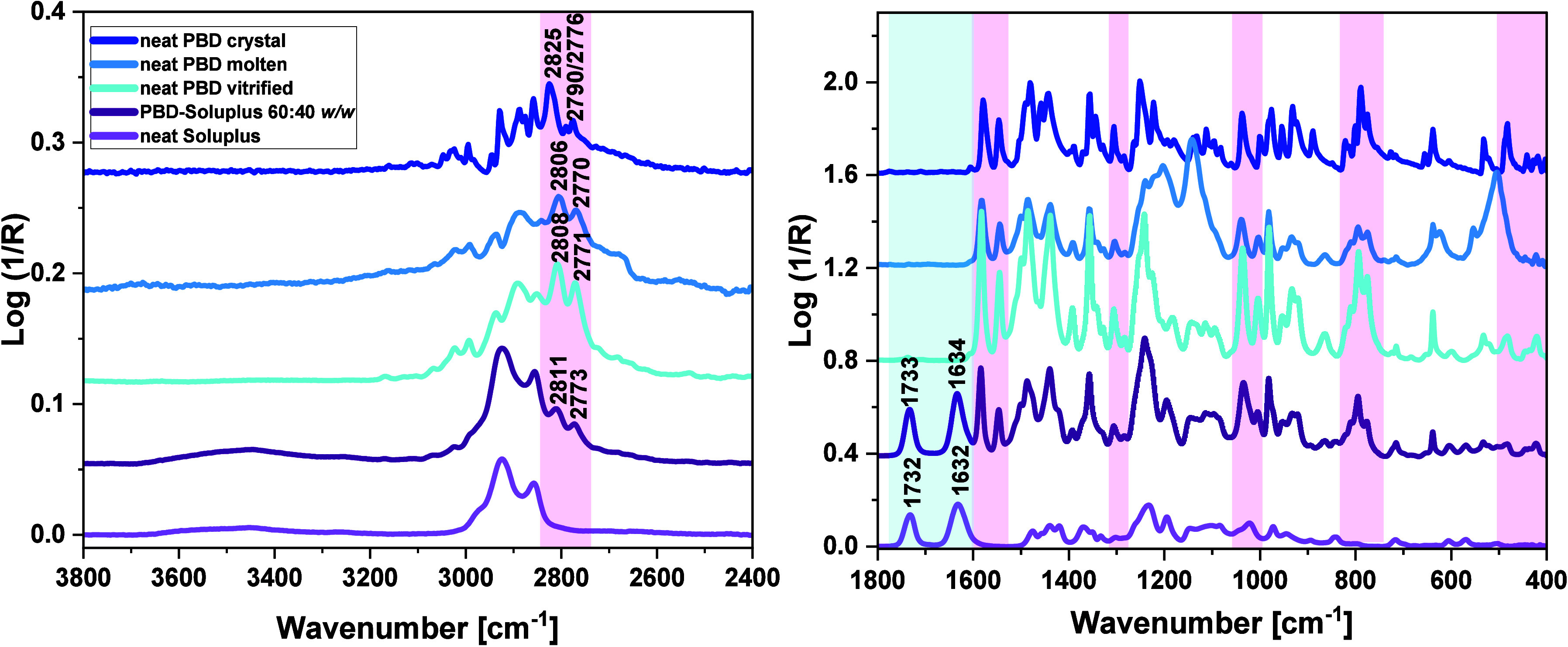
FTIR spectra of neat supercooled, crystal, and molten PBD as well as neat Soluplus and PBD-Soluplus 60:40 *w/w* BM, presented in the ranges of (left) 3800–2400 cm^–1^ and (right) 1800–400 cm^–1^. The spectral regions characteristic for neat PBD and neat polymer (not overlapped by the signals of the second compound) are highlighted in light pink and light blue, respectively.

**Figure 8:**
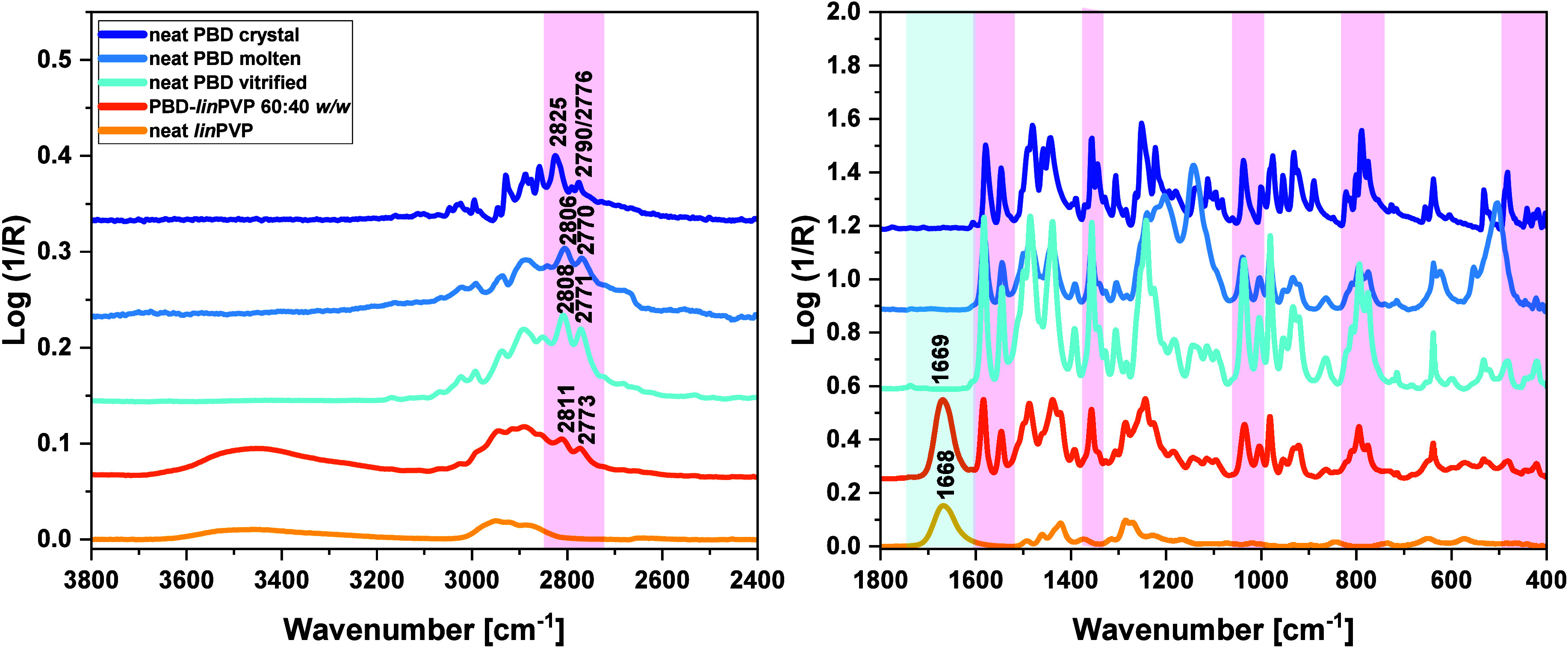
FTIR spectra of neat supercooled, crystal, and molten PBD as well as neat *lin*PVP and PBD-*linPVP* 60:40 *w/w* BM, presented in the ranges of (left) 3800–2400 cm^–1^ and (right) 1800–400 cm^–1^. The spectral regions characteristic for neat PBD and neat polymer (not overlapped by the signals of the second compound) are highlighted in light pink and light blue, respectively.

As depicted in Figures 7 and 8, the characteristic PBD peaks in PBD–polymer (Soluplus, *lin*PVP) mixtures exhibited only minor shifts in both the high- and low-wavenumber regions (highlighted in light pink). Specifically, the CH stretching peaks (*v*_*C*–*H*_) of 1,3-dioxolane ring of the neat PBD, located at 2808 and 2771 cm^–1^, are shifted to the higher wavenumbers (blue-shift) by approximately 2–4 cm^–1^ in the mixtures. Moreover, other PBD bands, located at lower wavenumber ranges of BMs, showed only slight shifts (ca. 1 cm^–1^) compared to those in neat vitrified API, as presented in Figure 9.

**Figure 9:**
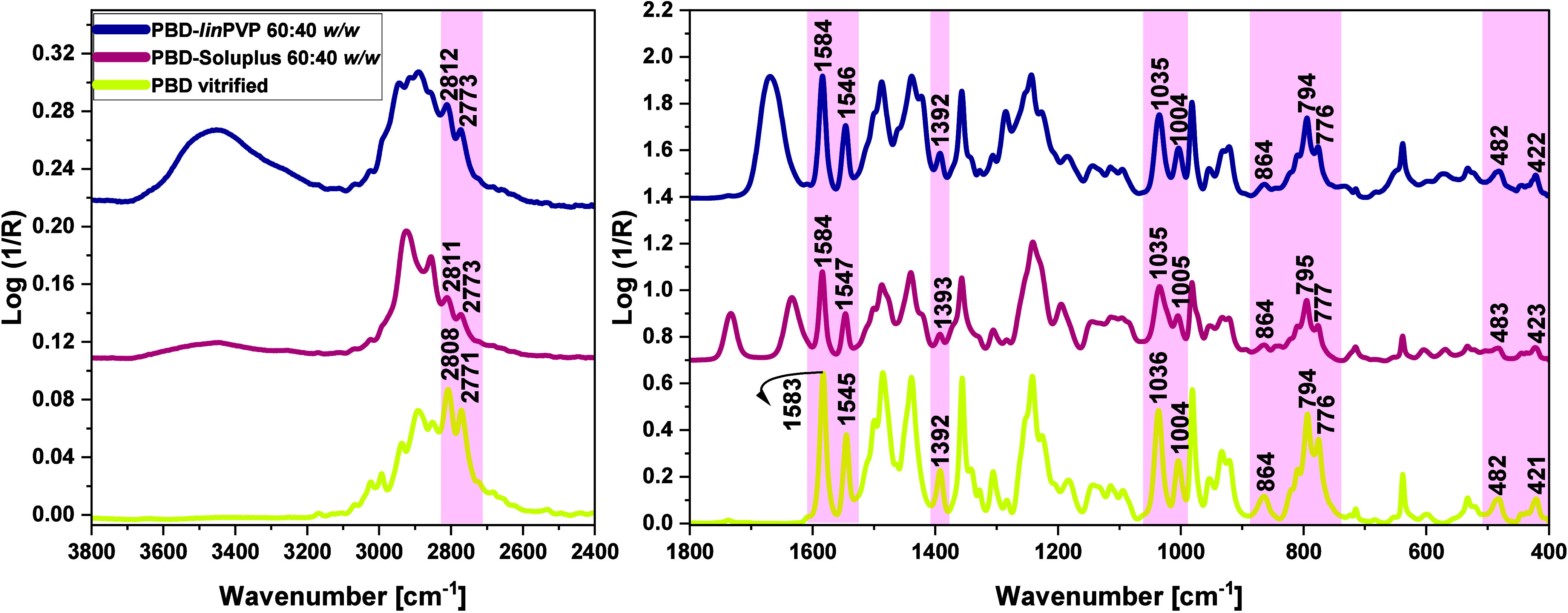
FTIR spectra of neat supercooled PBD as well as PBD-Soluplus and PBD-*lin*PVP 60:40 *w/w* BMs (fresh amorphous systems), presented in the ranges of (left) 3800–2400 cm^–1^ and (right) 1800–400 cm^–1^. The spectral regions characteristic for neat PBD (not overlapped by the signals of the polymer) are highlighted in light pink.

Notably, both Soluplus and *lin*PVP contain functional groups capable of interacting with the drug: Soluplus includes both proton-donating (−OH) and proton-accepting groups (ester carbonyl and tertiary amide carbonyl), while PVP contains a proton-accepting group (ester carbonyl). Therefore, the changes in peak positions associated with these functional groups of polymers after mixing with the neat API might suggest possible interactions between them. It is also worth mentioning that in the crystal, the C–H atoms of the PBD molecule (from the aliphatic – CH_2_ group and 1,3-dioxolane and pyrimidine rings) are involved in the weak *C*–*H···*π hydrogen bonds (HBs) with the pyrimidine and benzene rings as acceptors.^56^ These intermolecular interactions result in the blue shifts of the C–H bands of crystalline PBD compared to the corresponding CH peak positions in the molten PBD sample (Δ*v* = ca. + 20 cm^–1^), Figures 7 and 8.

To further verify this hypothesis, we analyzed the spectra of BMs, particularly in the regions corresponding to the stretching vibrations of the C=O groups (*v*_*C*=*O*_) of the studied polymers. As shown in Figure 7, the *v*_*C*=*O*_ bands of neat Soluplus located at 1732 cm^–1^ (the ester group, see **Figure S12** in the SI) and 1632 cm^–1^ (the tertiary amide group) exhibit minimal blue shift (shifted to higher wavenumbers) in the examined BMs (1733 cm^–1^, Δ*v* = +1 cm^–1^; 1634 cm^–1^, Δ*v* = +2 cm^–1^). Similarly, a very minor (insignificant) blue-shift is observed in the case of the *v*_*C*=*O*_ band in the API-*lin*PVP system (from 1668 cm^–1^ to 1669 cm^–1^, Δ*v* = +1 cm^–1^), Figure 8. These experimental facts indicate a lack of specific interactions (HBs or significant dipole–dipole interactions) occurring between the carbonyl groups of the studied polymers and API.

Overall, the most prominent shifts in the IR spectra of the BMs were observed for the C–H stretching bands of PBD (max +4 cm^–1^). Interestingly, the C–H peak position values of the API in BMs were essentially close to those detected in the IR spectrum of molten or vitrified PBD, in which some of the HBs involving weak *C*–*H*···π interactions were broken/disrupted. Thus, this observation implies that, in the studied binary systems, certain H-bonds between PBD molecules were destroyed by the introduction of PVP or Soluplus molecules (the steric hindrance). However, at the accuracy of our investigations, we can state that there are no differences in the intermolecular interactions between the API and the applied polymers. Hence, an argument that the higher physical stability of PBD in the BM composed of star or linear PVP, as deduced from DSC and XRD investigations, is related to the stronger intermolecular interactions between both components with respect to those occurring in the solid dispersions formed by Soluplus, can be easily rejected.

#### 3.1.4 BDS Data

According to previous DSC and XRD investigations, the self-synthesized PVP polymer matrices (both linear and branched) more effectively inhibit the recrystallization of PBD compared to the commercial Soluplus. As shown by FTIR studies, such differences in the crystallization behavior are not related to significant or specific interactions between the API and three examined macromolecules. Considering the results of XRD investigations, one can suppose that a greater physical stability of API-PVP BMs at room temperature with respect to API-Soluplus formulations is probably due to higher viscosities (η) related to greater *T_g_*’s of these systems (Figure 2). To confirm or exclude the effect of η on various stability of PBD-polymer mixtures, we decided to examine the molecular dynamics of all considered ASDs, especially to find such a *T*, at which the crystallization process will occur relatively fast and the viscosity of all systems will be constant. For this purpose, broadband dielectric spectroscopy (BDS) measurements in a wide temperature range, both above and below the *T_g_*, were carried out.

The loss spectra obtained for representative API-polymer 90:10 *w/w* ASDs are presented in Figure 10. Analogous data for PBD-polymer 80:20 *w/w* binary systems are illustrated in **Figure S13** in the SI. As shown in both figures, at *T* > *T_g_*, two processes can be identified in the spectra of each examined sample, both shifting toward lower frequencies (*f*) with decreasing *T*. The first one is a direct-current (*dc*) conductivity, associated with the transport of ionic impurities that are always present in the liquid. Meanwhile, the second process, located at higher *f,* is a structural (α) relaxation, originating from the cooperative motions of all molecules in the sample and responsible for the glass transition. At higher *T*, a decrease (subtle or more clear) in the amplitude of the α-peak can be detected, indicating the ongoing crystallization. On the other hand, in the glassy state (*T* < *T_g_*), two secondary relaxations (β and γ) with lower amplitudes dominate the spectra of ASDs. In all studied 90:10 and 80:20 *w/w* BMs, β- and γ- processes are fairly well separated from each other.

**Figure 10:**
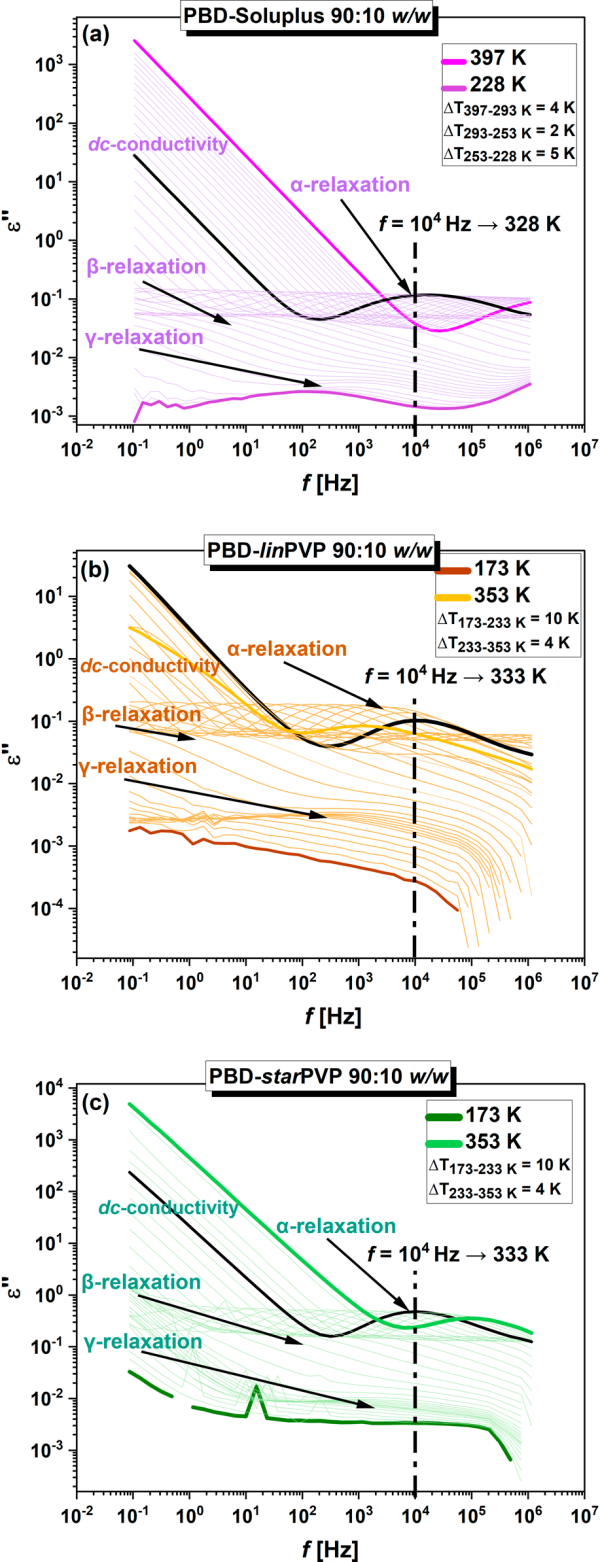
Dielectric loss spectra of **(a)** PBD-Soluplus, **(b)** PBD-*lin*PVP, and **(c)** PBD-*star*PVP 90:10 *w/w* binary mixtures collected at ambient *p* and in the indicated *T*-ranges.

It should be emphasized that the primary goal of molecular dynamics studies using BDS was not the in-depth analysis of the relaxation processes detected in the loss spectra of PBD-polymer systems (e.g., the estimation of *T_g_* or activation barrier for both secondary relaxations) but, as mentioned above, the determination of temperatures, at which different BMs exhibit similar viscosities. Herein, it is worth noting that according to Maxwell’s relationship,^70,71^ for a given/constant structural relaxation time (τ_α_ = 1/(2π*f*_α_)), a similar system viscosity (η) can be assumed. Based on this, we chose *T*, at which the maxima of α-peaks occur at *f* = 10^4^ Hz (see Figure 10 and **S13** in the SI, black spectra and black dash-dotted lines), hence τ_α_ is nearly the same. Next, isothermal crystallization studies at these temperatures (given in Table 1) and constant η/τ_α_ (isochoric conditions) were carried out for the examined binary formulations. Furthermore, it should be emphasized that to ensure comparable degrees of undercooling across all mixtures and to allow the analysis of crystallization kinetics based solely on the differences in the type of polymer used, the crystallization temperatures (*T*_*c*_) of each system had to be appropriately selected. **Table S3** in the SI presents the *T*_*g*(*DSC*)_ values for the 90:10 and 80:20 *w/w* ASDs, as well as the *T_c_*_*(BDS*)_ values for the corresponding systems. The $\frac{T_{c\left(BDS\right)}}{T_{g\left(DSC\right)}}$ ratios were then calculated, and it was found that they fall within a narrow range of 1.26–1.29 for all tested mixtures. This indicates that very similar degrees of undercooling were achieved. This is not surprising since all the crystallization studies were performed at isochronal conditions (the same viscosity). Therefore, differences in crystallization kinetics can be attributed to variations in the type and architecture of the polymer used in the ASD.

It should be added that to measure the progress of crystallization using the BDS method, we employed a standard approach – API and BMs were melted in the apparatus, supercooled, and then reheated to the appropriate crystallization temperatures (*T*_*c*_). The dielectric loss spectra obtained during the isothermal crystallization of PBD from representative 90:10 *w/w* systems are presented in Figure 11. The same data for PBD-polymer 80:20 *w/w* mixtures are given in **Figure S14** in the SI. As shown, in all cases, the amplitude of the α-relaxation process systematically decreases over time. A reason for this is the freezing of the molecular mobility during the ongoing crystallization.^72,73^

**Figure 11:**
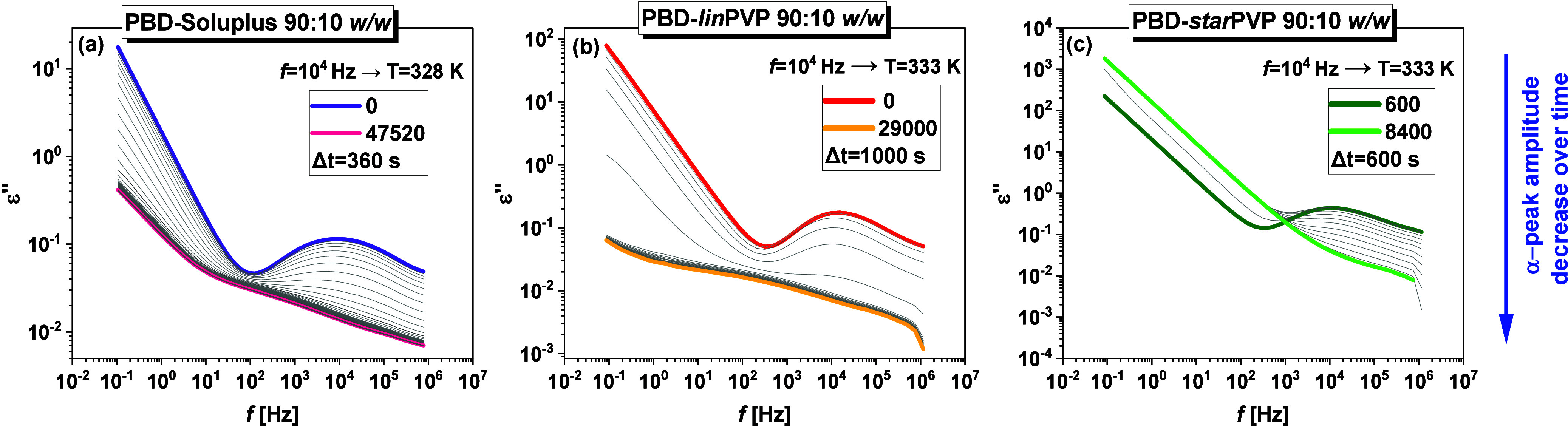
Time evolution of the imaginary (*ε* ^″^) part of the complex dielectric permittivity plotted versus frequency during the isothermal crystallization of **(a)** PBD-Soluplus 90:10 *w/w*, **(b)** PBD-*lin*PVP 90:10 *w/w*, and **(c)** PBD-*star*PVP 90:10 *w/w* at indicated temperatures (*f* = 10^4^ Hz).

To analyze the progress of this process in the studied BMs and neat API system, we followed the time dependency of the imaginary part of the complex dielectric permittivity (*ε* ^″^) measured at the frequency corresponding to the maximum of the α-peak. Next, *ε* ^″^ was normalized using the following equation

5 $$\varepsilon_{n}^{\prime \prime }\left(t\right)=\frac{\varepsilon^{\prime \prime }\left(0\right)-\varepsilon^{\prime \prime }\left(t\right)}{\varepsilon^{\prime \prime }\left(0\right)-\varepsilon^{\prime \prime }\left(\infty \right)}$$

where *ε* ^″^(0) is the *ε* ^″^ at the beginning of the crystallization, *ε* ^″^(∞) is the long-time limiting value, and *ε* ^″^(*t*) is the value at the time, *t*.

In Figure 12, the values of *ε*_*n*_^″^ are presented as a function of time. To analyze these data, the Avrami equation was applied

6 $$\varepsilon_{n}^{\prime \prime }=1-\exp\left(-kt^{n}\right)$$

where *k* is the rate constant of crystallization and *n* is the Avrami exponent (it is generally assigned to a dimension of growing crystals).^74^

**Figure 12:**
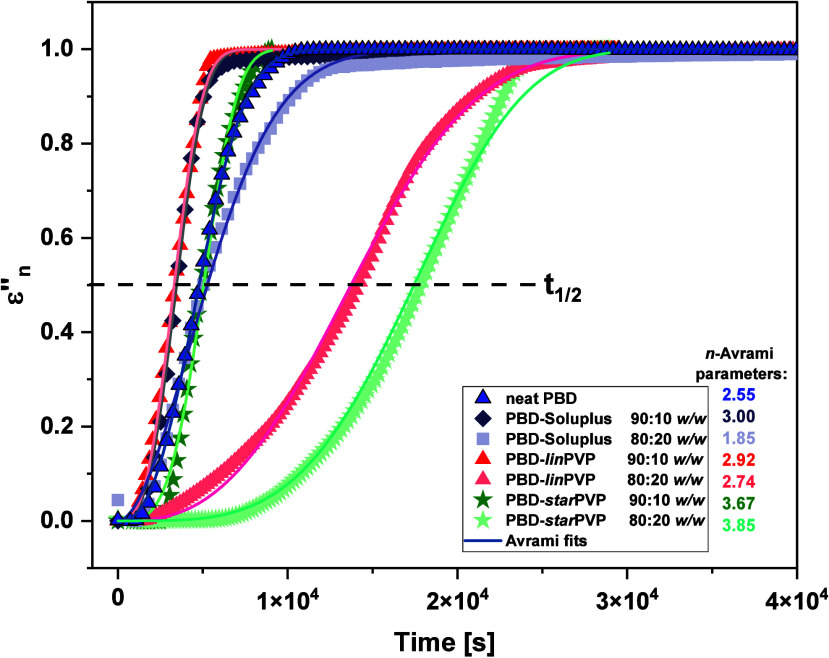
Time dependence of normalized permittivity *ε*_*n*_^″^ for neat PBD and API-polymer BMs in various ratios (90:10 and 80:20 *w/w*). The solid lines represent Avrami fits.

The solid lines in Figure 12 represent the best fits of eq 6 to experimental data, and as shown, the Avrami model describes them satisfactorily. The values of the parameters *k* and *n* are given in Table 1. From the analysis of *k*, it can be determined that the slowest crystallizing systems are PBD-*lin*PVP and PBD-*star*PVP 80:20 *w/w* (*k* = 6.37 × 10^–5^ and 5.21 × 10^–5^ s^–1^, respectively), while the fastest crystallizing ASDs are PBD-Soluplus 80:20 *w/w* and PBD-*star*PVP 90:10 *w/w* (*k* = 1.56 × 10^–4^ and 1.79 × 10^–4^ s^–1^, respectively). However, it should be remembered that the parameter *k* gives information on the crystallization rate, whereas the time scale of this process is also an extremely important aspect. To have insight into this property, we also analyzed the crystallization half-time (*t*_1/2_), which is the time required to reach 50% of the final crystallinity. It can be seen (Table 1) that the shortest *t*_1/2_ equal to 3 400 s was determined for both PBD-Soluplus and PBD-*lin*PVP 90:10 *w/w* mixtures. In contrast, PBD-*lin*PVP and PBD-*star*PVP 80:20 *w/w* mixtures were characterized by the longest *t*_1/2_ (=13 800 and 17 700 s, respectively), as well as the longest crystal nucleation time. The latter result corresponds well with those obtained from the analysis of *k* parameter (eq 6) as well as the outcomes of calorimetric and structural investigations.

In summary, the conducted studies showed that the differences observed in the stability of the amorphous PBD-polymer BMs are not related to the variations in the viscosity of the systems but rather to the architecture of the macromolecules. This conclusion is supported by dielectric studies performed at a constant η, which revealed entirely different crystallization behaviors of PBD depending on the polymer matrix used. Therefore, it can be stated that the type and topology of the polymer play a crucial role in PBD crystallization. Notably, hydrophilic PVP polymers, especially those with branched topologies and controlled macromolecular parameters, seem to be the most effective inhibitors of undesired API recrystallization from the amorphous form. However, it should be noted that once crystallization begins, it proceeds more rapidly in the BM (80:20 *w/w*) containing *star*PVP, as evidenced by the analysis of the crystallization rate constant.

### 3.2 Results of Experimental Studies on Micelles Composed of PBD and Various Polymers

Having comprehensively characterized the amorphous binary mixtures, the next step was to investigate in detail the impact of various polymer matrices on the physical stability of PBD and its release, which is directly related to its bioavailability and therapeutic effectiveness, using micellar systems.

#### 3.2.1 Micelle Formation and the Determination of Their Key Parameters

In the first step, before the preparation of PBD-polymer micelles, an important parameter - critical micelle concentration (CMC) - was determined for each polymer matrix. The CMC measurement is traditionally performed before micellar system formation to determine the exact macromolecule concentration at which micelles begin to form in solution. This concentration is crucial, as it marks the transition from the individual molecules to self-assembling aggregates (micelles), which exhibit significantly different physicochemical properties.^30^ The study of this key parameter was conducted for aqueous polymer solutions at various concentrations by analyzing changes in the surface tension as a function of solution concentration (see **Figure S15** in the SI). The detailed measurement procedure is described in the Materials and Methods section, while the determined CMC values for each polymer matrix are presented in Table 2. At this point, it is also important to highlight the structural differences among the studied polymers. The commercial macromolecule Soluplus is a typical amphiphilic polymer, containing both hydrophilic (PEG, PVAc) and hydrophobic (PCL) domains. In contrast, PVP polymers are distinctly hydrophilic; however, their structure is quite intriguing. Their main aliphatic carbon backbone is hydrophobic, while the cyclic butyrolactam groups (due to the presence of oxygen and nitrogen) exhibit strong hydrophilic properties.^75^ Therefore, despite the lack of typical amphiphilicity (i.e., the absence of two distinct hydrophilic and hydrophobic polymer segments), PVP polymers may still have a good tendency to form micellar systems. Thus, determining the CMC value is an important and highly interesting aspect, as its value can vary significantly depending on factors such as polymer topology and chain length (due to steric effects), as well as the presence of (non)­polar domains (structural effects).^76^ As presented in Table 2, the CMC values follow the trend: *star*PVP < Soluplus < *lin*PVP. Scientific data report that the CMC is generally lower for compounds with a higher degree of hydrophobicity and higher for substances with greater hydrophilicity. Additionally, the longer the hydrophilic block, the higher the CMC values, indicating a lower tendency to form micelles. This is because, as the polymer chain length increases, the number of hydrogen bonds between the repeating units of the hydrophilic block and water molecules also increases, resulting in greater water solubility and a higher CMC value.^77,78^ Thus, it should be noted that our data correspond well with literature reports. The macromolecule *lin*PVP (with the longest hydrophilic chain) exhibits a slightly higher CMC value compared to the commercial copolymer Soluplus, which has a somewhat shorter hydrophilic segment. On the other hand, when comparing linear and star-shaped samples, the *star*PVP demonstrates a significantly lower CMC value than its linear counterpart. This is likely due to the presence of three shorter hydrophilic arms/blocks, leading to a higher density of PVP segments that facilitate the self-assembly process. Therefore, it has been concluded that star-shaped polymers are more prone to micelle and aggregate formation than linear ones, which is also consistent with findings from other literature reports.^79^

**Table 2:** Parameters Describing the Tested Micellar Systems

***No.***	***Micellar systems***	***CMC*** [μg/mL]	***DLC*** [%]	***DLE*** [%]	***d***_***h***_**_(TEM)_** [nm]	***d***_***h***_**_(DLS)_** [nm]	***ZP*** [mV]
1.	PBD-Soluplus 1:1 (5:95)	95	4.26	3.72	162.41	67.09	–9.20
2.	PBD-Soluplus 1:2 (5:95)		4.57	8.31	164.70	61.82	–7.23
3.	PBD-*lin*PVP 1:1 (2:98)	105	2.06	1.74	104.07	46.02	–16.17
4.	PBD-*lin*PVP 1:2 (2:98)		2.17	3.46	86.03	39.47	–12.60
5.	PBD-*star*PVP 1:1 (5:95)	65	4.84	4.36	684.70	174.75	–13.53
6.	PBD-*star*PVP 1:2 (10:90)		9.76	16.21	660.80	134.40	–11.90

After determining the CMC parameter, micelles were prepared at two weight ratios (1:1 and 1:2) following a well-described and widely used method in the literature.^80^ The procedure for obtaining micellar systems based on PBD and various macromolecules (Soluplus, *lin*PVP, *star*PVP) is detailed in the Materials and Methods section. After successful preparation, UV–Vis measurements were performed on the neat API (see **Figures S16** and **S17** in the SI) as a reference sample. This step was essential to subsequently assess the encapsulation efficiency of PBD and determine two parameters – drug loading efficiency (DLE) and drug loading content (DLC), which are crucial in the design, evaluation, and optimization of DDSs. DLE (often also called encapsulation efficiency) represents the percentage of drug successfully incorporated into the DDS compared to the total amount of the drug initially used during the formulation process. On the other hand, DLC describes the amount of API present in the DDS relative to the total weight of the drug carrier.^81^ DLE and DLC values for all tested micellar systems, calculated according to eqs 1 and 2, respectively, in the Materials and Methods section, are shown in Table 2. As can be seen, the values of DLE remain relatively low in all cases, ranging from 1.74% to 16.21%. Similarly, the values of DLC also fluctuate at relatively low levels, not exceeding 10%. For instance, in the PBD-Soluplus and PBD-*lin*PVP micellar systems, they were approximately 4% and 2%, respectively, indicating that only a small fraction of the added PBD was successfully encapsulated. In contrast, the PBD-*star*PVP system exhibited a slightly higher DLC compared to the other systems. Moreover, a noticeable difference was observed between the 1:1 (4.84%) and 1:2 (9.76%) formulations. Additionally, the PBD-*star*PVP 1:2 system demonstrated the highest DLE of 16%. Therefore, it was concluded that the micellar system incorporating the star-shaped polymer matrix exhibits the most favorable DLC and DLE parameters. It should be noted that the initial amounts of drug and polymers used differ significantly from the actual amount of drug encapsulated in the polymeric matrix. Therefore, due to the considerable discrepancies between the initial and final drug–polymer ratios in the micellar systems, both the initial ratio and (in brackets) the actual content of API and polymer in each formulation are provided throughout the following sections of this study. These two ratios are also presented in Table 2 (outside the brackets – initial ratio; in brackets – actual drug-polymer ratio after the micellization process).

The obtained drug-loaded micellar systems were further analyzed using Dynamic Light Scattering (DLS). This technique is used to determine the hydrodynamic diameter (*d*_*h*_) of micelles, providing insight into their size distribution and aggregation behavior in solution.^82,83^ As observed in **Figure S18** in the SI, and based on the *d*_*h*_ values presented in Table 2, the micelles’ hydrodynamic diameters vary depending on the polymer matrices used, following the trend: PBD-*lin*PVP < PBD-Soluplus < PBD-*star*PVP. Furthermore, as can be clearly observed in Figure S18a, Soluplus seems to be the best candidate to form micelles with PBD since the size distribution of the self-assembles in this case was the lowest of all studied systems. In contrast, in the remaining cases (PVP-based micelles), two fractions of micellar systems with different sizes can be observed (Figure S18bc). Additionally, DLS measurements allowed for the determination of the Zeta Potential (*ZP*), which helps assess the surface charge of micelles and its impact on their colloidal stability. A low negative *ZP* value indicates strong electrostatic repulsion between particles, preventing aggregation.^84^ Values closer to zero suggest lower stability, as weaker repulsive forces increase the likelihood of micelle coalescence or precipitation. Thus, measuring the *ZP* provides insight into the colloidal stability and shelf life of micellar systems. Based on our data analysis, the most stable micellar systems were PBD-*lin*PVP and PBD-*star*PVP, exhibiting similar *ZP* values of approximately −12 mV. In turn, the PBD-Soluplus system was less stable and exhibited a higher *ZP* value (−7.23 mV).

In this study, we also visualized the microstructure of the investigated micellar systems using Transmission Electron Microscopy (TEM). As seen in Figure 13, the obtained structures are well-defined and suggest that the drug migrates into the core of the formed particles, surrounded by polymer chains. The observed spherical structures further confirm the micellar architecture. It is worth noting that the three different micellar systems vary in *d*_*h*_, as determined by TEM. Therefore, in the next step, micellar particle size statistics were conducted (Figure 14 **)**, and the average *d*_*h*_ values are presented in Table 2. Based on TEM measurements, a similar trend was observed as in DLS measurements, i.e., the PBD-*lin*PVP system exhibited the smallest *d*_*h*_ values, while PBD-*star*PVP – the largest. However, a significant discrepancy in *d*_*h*_ parameters was noted between the two independent methods (DLS vs. TEM). This difference may result from the sample preparation process for TEM measurements. Indeed, solvent evaporation can considerably increase the material concentration, potentially leading to aggregation or forming of larger particles. It is important to highlight that similar phenomena have also been observed in other micelle-related studies.^76^

**Figure 13:**
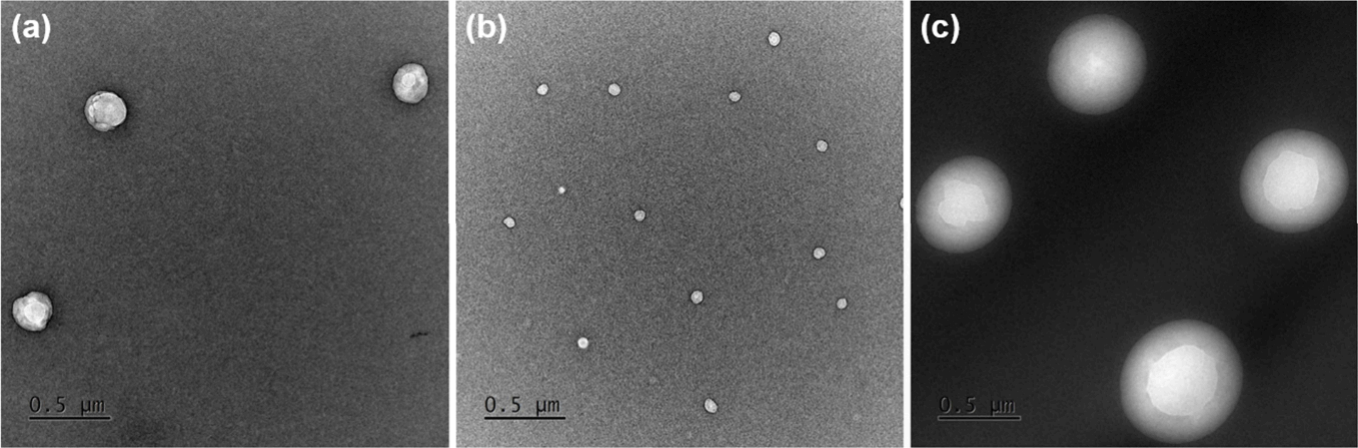
TEM images of **(a)** PBD-Soluplus micelles 1:2(5:95), **(b)** PBD-*lin*PVP micelles 1:2(2:98), and **(c)** PBD-*star*PVP micelles 1:2(10:90). Scale = 0.5 μm.

**Figure 14:**
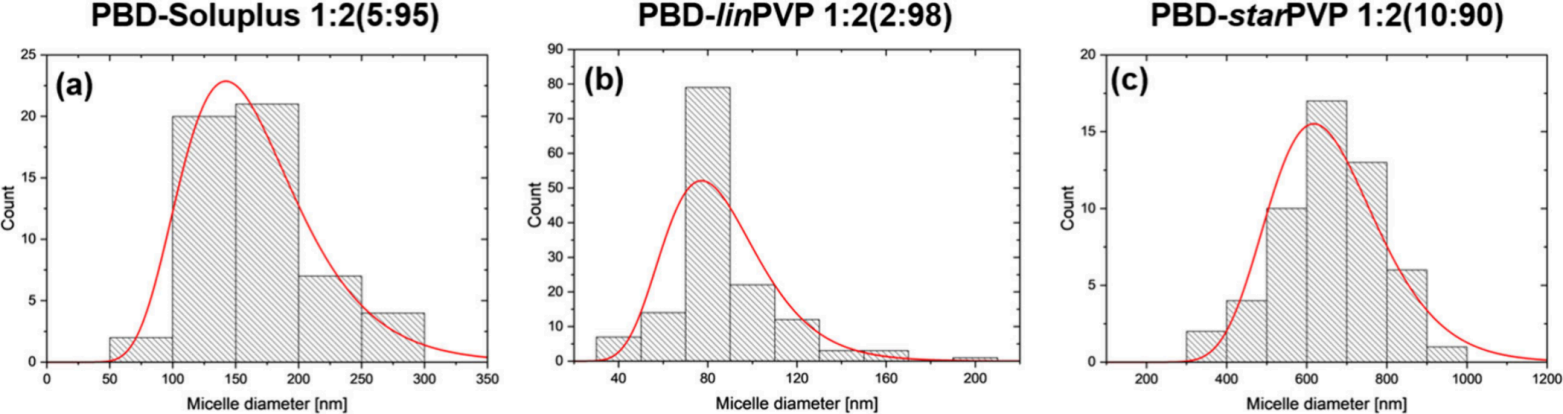
Size distribution of obtained micelles based on collected TEM data for **(a)** PBD-Soluplus, **(b)** PBD-*lin*PVP, and **(c)** PBD-*star*PVP micellar systems.

#### 3.2.2 XRD Data

Subsequently, to characterize the stability of PBD in its amorphous form when encapsulated in polymeric micellar systems, structural (XRD) measurements were performed. Figure 15, respectively, show the XRD patterns of PBD-Soluplus BMs and PBD–PVP micellar systems (both freshly prepared samples and those stored for 21 days at room temperature). Moreover, in Figure 15a, the structural data determined for the amorphous PBD-Soluplus 5:95 *w/w* BM, which contains approximately the same amount of loaded API as the PBD-Soluplus 1:2 micellar system, are presented. As can be seen, both PBD-Soluplus (1:1(5:95) and 1:2(5:95)) and PBD-*lin*PVP (1:1(2:98) and 1:2(2:98)) were amorphous just after preparation and remained stable in this form after 3 weeks of storage at 293 K. In turn, the active substance in PBD-*star*PVP micellar systems (1:1(5:95)) recrystallized immediately (to the polymorphic form I), hence it was impossible to obtain it in the stable amorphous form.

**Figure 15:**
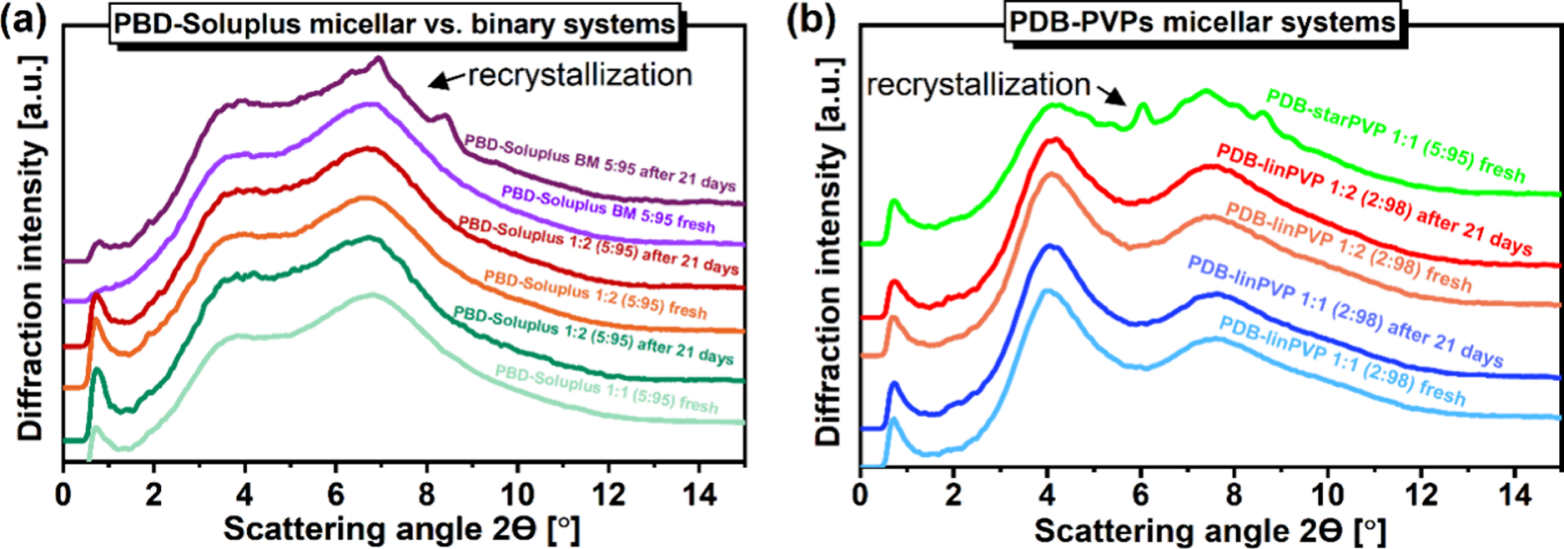
XRD patterns of **(a)** freshly prepared and stored by 3 weeks PBD-Soluplus micellar systems and PBD-Soluplus 5:95 *w/w* amorphous binary mixture as well as **(b)** PBD–PVPs micellar systems.

Moreover, when we compare the ASD and the micellar system with the same PBD content (PBD-Soluplus 5:95 *w/w* and 1:2(5:95), respectively), one can state that (*i*) both fresh samples were amorphous, (*ii*) the more stable was the latter, i.e., the micellar system, (*iii*) in the case of 5:95 ASD, the recrystallization of PBD during 3 weeks of storage was observed. However, the recrystallized phase may be a new polymorphic form since the system of Bragg peaks is different than that for polymorphic form I.

### 3.3 Drug Release Studies

At the final stage, we carried out the studies of PBD release from various API-polymer ASDs and micelles. It is important to emphasize that such investigations are crucial in the aspect of developing new macromolecules as effective drug carriers and, consequently, more efficient DDSs. It is also worth noting the significant challenges related to the bioavailability of the given pharmaceutical. In this context, it should be stressed that the use of PBD is significantly limited by its very low (<10%) oral bioavailability due to extensive first-pass hepatic metabolism and a short biological half-life. As a result, the drug is prescribed with a high dosing frequency of 3–5 tablets per day.^85,86^ Nevertheless, it should be clearly emphasized that although first-pass hepatic metabolism is the main factor contributing to the poor bioavailability of PBD, approximately 50% of the drug is eliminated from the body within 24 h (according to Servier – Summary of Product Characteristics for Pronoran). Therefore, improving the solubility of PBD using appropriate excipients may potentially increase the amount of API absorbed in the intestines and subsequently entering systemic circulation. A higher absorbed drug dose could enhance therapeutic efficacy, as hepatic enzymes would require more time to metabolize a larger amount of the active substance. Moreover, research performed over the past two decades has shown that, in addition to improving motor symptoms, PBD has the unique advantage of alleviating nonmotor symptoms, such as apathy, or cognitive impairment. This has renewed interest in this API, which may enhance its clinical application in the future.^86–88^ Due to the highly inefficient current oral route for PBD delivery,^89^ the ongoing search for novel polymer matrices and their application in various DDSs represents a very important and intriguing area of research.

The PBD release tests were carried out using a special FaSSIF medium at pH = 6.5. At this point, it is important to justify the selection of the dissolution medium. PBD exhibits pH-dependent solubility – it is highly soluble under gastric (acidic) conditions but poorly soluble under intestinal (near-neutral) conditions (*pK*_*a*_ *=* 6.94).^90^ However, the intestines are the primary site of drug absorption. Therefore, evaluating the solubility of PBD under intestinal conditions appears to be a rational strategy for properly assessing the potential improvement in drug absorption. The aim of the dissolution studies was primarily to assess how various excipients in the formulations influence both the drug release kinetics and the saturation solubility. Since saturation solubility must be determined under stable conditions where the drug remains in equilibrium, a 24-h time frame is commonly used, although longer periods may be applied for drugs with slower dissolution rates. In our study, we adopted a measurement time of up to 24 h. Under these conditions, it was assumed that both the drug and the polymeric matrices were in a stable state. According to forced degradation studies by Kumar et al., PBD is not susceptible to acid, base, or water hydrolysis.^91^ Moreover, HPLC analysis (used to determine the concentration of released drug) did not reveal any additional peaks that could be attributed to degradation products of API. On the other hand, the applied PVP polymers are not pH-sensitive, and the FaSSIF medium used for the release studies is a buffered solution. This allows us to reasonably assume that the pH of the medium remained largely stable throughout the dissolution process, with only minimal potential fluctuations.

The first tests involved the release of the API from ASDs (Figure 16). It should be noted that the release profiles presented in Figures 16 and 17 refer to the percentage of drug released, whereas in the SI, we showed the drug concentration (mg/mL) vs time (Figures S19 and S20). As can be observed in Figure 16, neat crystalline PBD achieves a drug release level of about 30% after 60 min of the test, and then the solubility changes (increases) only slightly with time. Moreover, there are small differences in the release profiles of crystalline PBD and that used in the commercial formulation (called Pronoran, which mainly contains the API, commercial PVP/Povidone and talc). However, as clearly seen, for each investigated API-polymer ASD, a noticeable improvement in the API release from the matrix compared to the neat crystalline PBD can be observed. Increasing the macromolecules’ content generally results in a higher amount of released drug. Note that similar conclusions were drawn in our previous studies on itraconazole-PVP BMs.^51^ It is also worth noting that all conducted release tests showed similar behavior: for approximately 60–100 min (or ∼ 200 min in the case of PBD-Soluplus 80:20 *w*/*w*), an intense release of the API from the polymer matrices was observed. During this period, the drug was rapidly released and dissolved until reaching a supersaturated state. However, after this time, most tests revealed a decrease in the amount of dissolved amorphous PBD, suggesting the onset of precipitation. Such observations suggest the presence of the so-called “parachute effect”. Initially, the drug dissolves relatively quickly, but then there is a tendency for it to precipitate due to exceeding its equilibrium solubility. However, thanks to the stabilizing properties of the polymers, PBD remains in a metastable supersaturated state, preventing or delaying crystallization – effectively slowing down the “fall” (precipitation). This effect is often considered highly beneficial, as it can extend the absorption window in the gastrointestinal tract, thereby potentially increasing the oral bioavailability of poorly water-soluble drugs.^92–94^ Additionally, Figure 16d compares the release profiles for the 60:40 *w/w* ASDs containing different polymer matrices. This comparison clearly indicated that the most favorable formulation (showing the highest amount of released drug, ∼ 50%) is the PBD-Soluplus 60:40 *w/w* ASD. Moreover, keeping in mind the discovery of a new polymorphic form of PBD, an interesting aspect was to verify whether the polymorph II remains stable during the drug release studies from the binary formulations. Therefore, additional calorimetric measurements were performed to identify the existing polymorphic forms of the drug in one selected, representative formulation (PBD–*lin*PVP 80:20 *w/w*), both before and after the release process. As clearly shown in Figure S21 in the SI, the sample prior to the release exhibited two endothermic peaks assigned to melting at *T_m_* *_(II)_* = 362 K and *T*_*m*(*I*)_ = 369 K, indicating the presence of both polymorphs (I and II). In contrast, the same sample analyzed after the release process revealed only a single, strong endothermic event corresponding to the melting of polymorphic form I of PBD (*T*_*m*(*I*)_ = 371 K). Thus, the calorimetric studies demonstrated that new polymorph II of the API converts to the primary polymorph I upon contact with the release medium/solution.

**Figure 16:**
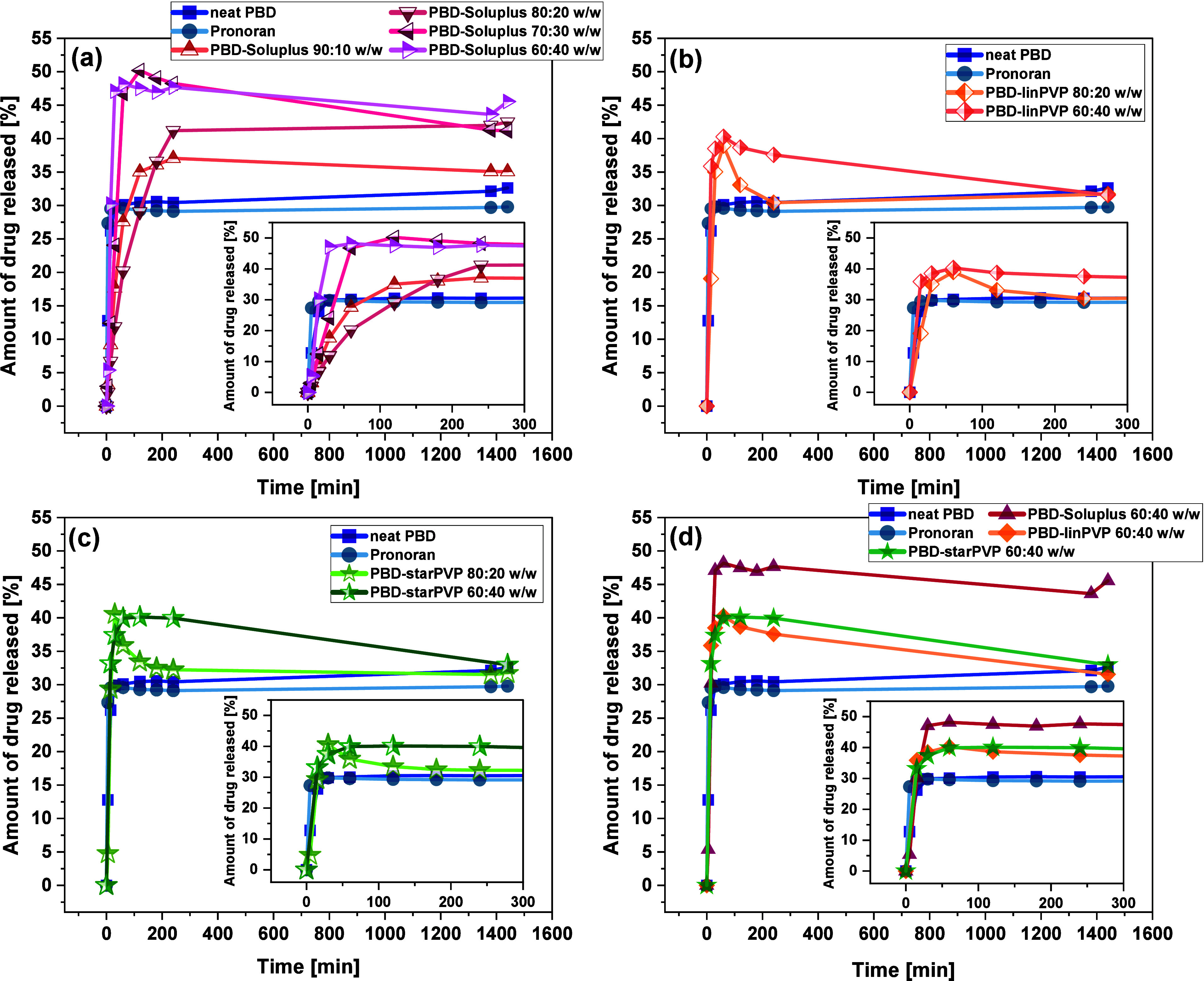
Drug release profile (percentage of drug released vs. time) from various polymer matrices in BMs: **(a)** PBD-Soluplus, **(b)** PBD-*lin*PVP, and **(c)** PBD-*star*PVP. Panel **(d)** presents a comparison of drug release profiles from various polymer matrices for 60:40 *w/w* BMs. Each panel contains two reference samples: neat crystalline PBD and the API Pronoran. The insets in each panels present the API release profiles over a shorter time (up to 300 min), which highlights the differences in the release kinetics.

**Figure 17:**
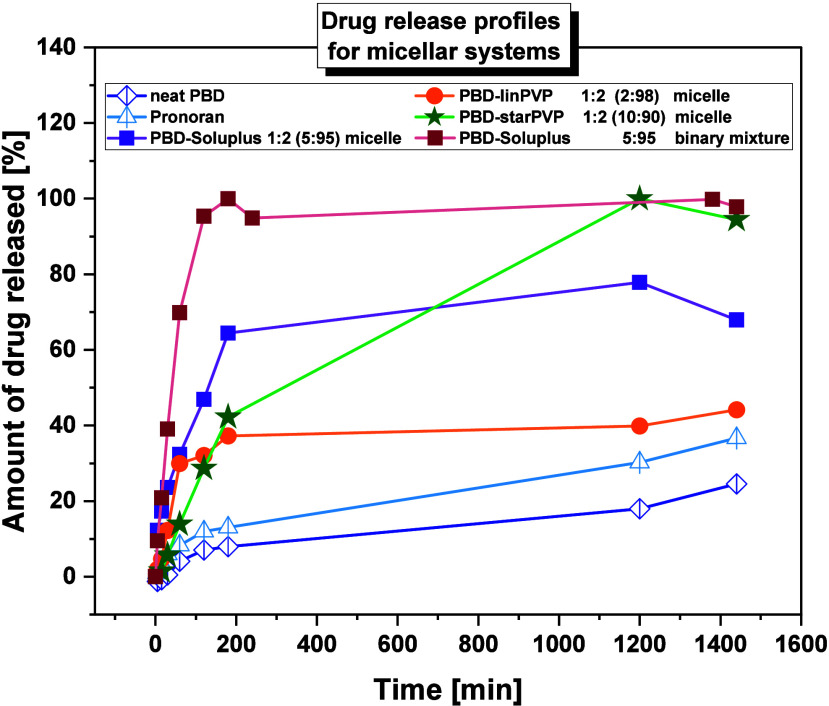
Drug release profiles (percentage of drug released vs. time) for micellar systems with various polymer matrices, as well as for the reference samples: neat PBD and the Pronoran tablet.

Analogous release tests were then conducted for the 1:2 API-polymer micellar systems. Notably, despite the relatively small amounts of drug successfully encapsulated (see DLC values in Table 2), it was possible to precisely determine the API release profile, as shown in Figure 17. At this point, it is worth adding that alongside the micellar systems, the PBD-Soluplus 5:95 *w/w* BM was also tested, which, as mentioned earlier, in terms of the API content, is similar/analogous to the PBD-Soluplus micellar system. As can be observed, the most desirable release profile is exhibited by the PBD-*star*PVP 1:2 (10:90) micelle. In contrast to other systems, where a rapid API release (for around 200 min) and then slow changes up to the end of the test are observed, herein a gradual API release (up to 99%, ∼ 1 mg/mL) over 1200 min/20 h, which represents the most favorable sustained release behavior, is visible. On the other hand, it is important to highlight the comparison between the micellar system and the binary mixture with approximately the same API content (∼5 wt %). The noticeably higher drug release (∼99%, ∼ 1 mg/mL) from the PBD-Soluplus 5:95 *w/w* BM compared to the PBD-Soluplus 1:2 (5:95) micellar system (∼70%, ∼ 0.7 mg/mL) clearly indicates better bioavailability of the API contained in the ASD. Such findings emphasize the very important aspect of these studies, i.e., the testing of new polymeric drug carriers in various types of formulations.

Summarizing, the formation of all PBD-polymer ASDs resulted in improving API release in the FaSSIF medium with respect to the crystalline substance (30%). The most visible effect was observed for the BMs with Soluplus (an increase even up to 50%). Moreover, the PBD-Soluplus 1:2 (5:95) micellar system was characterized by better solubility in FaSSIF than the ASD with the same polymer content (5:95 *w*/*w*). Among all 1:2 micelles, the best/the more favorable release profile (a gradual API release over 20 h up to 99%) was determined for the formulation with *star*PVP. In other cases, we observed a fast dissolution of the sample (within the first 200 min of the test), followed by small changes in the API content. In the case of PBD-polymer (90:10–60:40 *w*/*w*) ASDs, a large amount of PBD was released during the first 60–100 min of the experiment.

## 4 Conclusions

In this paper, several experimental techniques were applied to study ASDs and micellar systems composed of the low water-soluble and bioavailable API – piribedil and three polymer matrices differing in structure and properties: the commercially available Soluplus, self-synthesized linear PVP (*lin*PVP), and self-synthesized three-arm star-shaped PVP (*star*PVP). Particular attention was devoted to examining the influence of polymer type, topology, and its amount in the system on inhibiting the recrystallization of PBD from the amorphous BMs, changes in phase transition temperatures, and the potential enhancement of API’s pharmacokinetic properties.

A highly intriguing discovery was made at the initial stage of our research. Specifically DSC and XRD studies revealed that in PBD–polymer ASDs, the API recrystallizes primarily (API-PVP) or solely (API-Soluplus BMs with the polymer content ≥ 20) into a completely new polymorphic form II exhibiting *T*_*m*(*II*)_ ∼ 363 K, which is lower than the melting temperature of polymorph I (*T*_*m*(*I*)_ ∼ 370 K). Furthermore, XRD, nonisothermal DSC and isothermal BDS measurements indicated that the amorphous PBD is the most physically stable in ASDs with hydrophilic self-synthesized PVP macromolecules. However, among the PVP matrices, the star-shaped polymer demonstrated a slightly stronger ability to suppress the recrystallization of PBD from the amorphous phase compared to its linear counterpart. FTIR and BDS studies confirmed that there are no specific API-polymer interactions and variations in the system viscosity which can influence the observed differences in the crystallization behavior of PBD–PVP and PBD–Soluplus ASDs. It was stated that the factor affecting the physical stability of the examined binary systems is the architecture/topology of macromolecules.

We also showed that the commercially available amphiphilic copolymer Soluplus is more suitable for forming micellar systems. Only in the case of PBD–Soluplus micelles, a low/homogeneous particle size distribution was observed. Moreover, as revealed by XRD studies, in these micellar systems (and in those with *lin*PVP), PBD remained in the amorphous form for a longer period than in the micelles containing *star*PVP. What is more, PBD-Soluplus 1:2(5:95) micelle in comparison to PBD-Soluplus ASD with the same API content (5:95 *w*/*w*) was more physically stable.

Finally, drug release measurements indicated that the amount of PBD released from all tested formulations in a mildly acidic environment (FaSSIF medium, pH = 6.5) is clearly enhanced compared to the neat crystalline API (∼30%). Notably, the most significant improvement in solubility was observed for PBD–Soluplus 60:40 *w/w* ASD (∼50%) and the PBD–*star*PVP 1:2 (10:90) micellar system (∼99%). Importantly, for the latter formulation, the most desirable release profile (a gradual API release over 20 h instead of a fast API release within the first 1–3.5 h) was observed. Therefore, it can be assumed that the use of different polymer matrices as novel drug carriers in various types of formulations is an interesting perspective. Macromolecules can significantly contribute to improving the solubility of PBD as well as other poorly soluble APIs, ultimately increasing their bioavailability. From the pharmaceutical sector’s perspective, the results of our studies are highly promising. We believe they open a new pathway in the scientific discussion regarding the search for new polymorphic forms of APIs and the impact of polymer type, topology, as well as the kind of formulation, in the context of the enhancement of the bioavailability of numerous drugs.

## Supplementary Material



## Notes

Information on the synthetic pathways of *lin*PVP and *star*PVP together with the description of Size Exclusion Chromatography (SEC) and Nuclear Magnetic Resonance (NMR); TGA traces of neat API and polymers; ^1^H NMR spectra of *lin*PVP and *star*PVP; A comparison of ^1^H NMR spectra of the binary and physical PBD-Soluplus and PBD-*lin*PVP 60:40 *w/w* mixtures; The calorimetric *T_g_* values (ϕ = 10 K/min) for the examined PBD-polymer BMs as well as neat polymers in various weight ratios; The calorimetric *T*_c_ values for neat PBD and PBD-polymer 90:10 and 80:20 *w/w* mixtures depending on the ϕ; DSC thermogram (ϕ = 10 K/min) for PBD-*lin*PVP 60:40 *w/w* BM heated up to 473 K; DSC thermograms of PBD-Soluplus and PBD-*lin*PVP BMs (90:10, 80:20, 70:30, 60:40 *w/w*) at a heating rate of 2 K/min; Graphs of $\left(\frac{1}{T_{m}^{mix}}-\frac{1}{T_{m}^{pure}}\right)\left(\frac{{\Delta H}_{fus}}{-R}\right)-\ln\phi_{drug}-\left[1-\left(\frac{1}{m}\right)\right]\phi_{polymer}$ vs. ϕ_*polymer*_^2^ to determine the χ values for PBD-Soluplus and PBD-*lin*PVP systems; a brief description of the F–H theory and the method of determining the miscibility of binary systems; DSC thermograms collected at ϕ = 10 K/min for neat polymers as well as for PBD-Soluplus 80:20 and 70:30 *w*/*w* systems after recrystallization at room temperature; FTIR spectra of PBD-*lin*PVP and PBD-*star*PVP 60:40 *w/w* BMs, presented in the ranges of 3800–400 cm^–1^; FTIR spectra of crystalline PBD as well as Soluplus and *lin*PVP samples, measured in the spectral regions 3800–2400 cm^–1^ and 1800–400 cm^–1^ and their short description; Dielectric loss spectra of PBD-Soluplus, PBD-*lin*PVP, and PBD-*star*PVP 80:20 *w*/*w* BMs; Time evolution of *ε* ^″^ plotted versus *f* during the isothermal crystallization of the examined PBD-polymer 80:20 *w/w* systems at indicated temperatures (*f* = 10^4^ Hz); Determination of CMC by measuring the surface tension of serial dilutions of Soluplus, *lin*PVP, and *star*PVP; UV–Vis spectra for PBD at different concentrations (in the range of 2–15 μg/mL; solutions in ethanol solvent); the calibration curve for PBD in ethanol; Hydrodynamic diameters of the obtained PBD-Soluplus, PBD-*lin*PVP, and PBD-*star*PVP 1:2 micellar systems; Drug release profile (drug concentration vs. time) from various polymer matrices in binary mixtures and micellar systems; and DSC thermograms (ϕ = 10 K/min) for the PBD–*lin*PVP 80:20 *w/w* BM before and after the release process (PDF)
