# Supplementary material for: Hydrophilic and Amphiphilic Macromolecules as Modulators of the Physical Stability and Bioavailability of Piribedil: A Study on Binary Mixtures and Micellar Systems
Source: Mol Pharm. 2025 Jun 30;22(8):4708–30. doi: 10.1021/acs.molpharmaceut.5c00276 (PMC12326366; doi:10.1021/acs.molpharmaceut.5c00276)
Supplement: Supplementary file 1 [file mp5c00276_si_001.pdf]

Supporting Information

Hydrophilic and Amphiphilic Macromolecules as Modulators of the Physical Stability and Bioavailability of Piribedil: A Study on Binary Mixtures and Micellar Systems

Luiza Orszulak[1]*, Aldona Minecka[2], Roksana Bernat[3], Taoufik Lamrani[4], Karolina Jurkiewicz[4], Barbara Hachuła[1], Magdalena Tarnacka[4], Monika Geppert-Rybczyńska[1], Maciej Zubko[3,5], Marcela Staniszewska[6], Michał Smoleński[6], Justyna Dobosz[6], Grzegorz Garbacz[6], Kamil Kamiński[4], Ewa Kamińska[2]

[1] *Institute of Chemistry, Faculty of Science and Technology, University of Silesia in Katowice, Szkolna 9, 40-006 Katowice, Poland*

[2] *Department of Pharmacognosy and Phytochemistry, Faculty of Pharmaceutical Sciences in Sosnowiec, Medical University of Silesia in Katowice, Jagiellonska 4, 41-200 Sosnowiec, Poland*

[3] *Institute of Materials Engineering, University of Silesia in Katowice, 75 Pulku Piechoty 1A, 41-500 Chorzow, Poland*

[4] *Institute of Physics, Faculty of Science and Technology, University of Silesia in Katowice, 75 Pulku Piechoty 1A, 41-500 Chorzow, Poland*

[5] *Department of Physics, Faculty of Science, University of Hradec Králové, Rokitanského 62, 500 03, Hradec Králové, Czech Republic*

[6] *Physiolution Polska sp. z o.o., Skarbowcow 81/7, 53-025 Wroclaw, Poland*

**Corresponding author:* luiza.orszulak@us.edu.pl

Experimental

1. *Synthetic pathway of PVP with linear topology (linPVP)*

Thermally-initiated Reversible Addition Fragmentation Chain Transfer (RAFT) polymerization of *N*-vinylpyrrolidone (VP) using CTA1 (cyanomethyl methyl(4-pyridyl) carbamodithioate) as a chain transfer agent and 2,2'-azobis(2-methylpropionitrile) (AIBN) as an initiator with molar ratios $[VP]_0/[CTA1]_0/[AIBN]_0 = 1300/1/0.25$) was carried out as follows. Prior to polymerization, VP was passed through an alumina column to remove the inhibitor. CTA1 and VP were placed in a Schlenk flask with a magnetic stirring bar. The solution was purged under nitrogen and purified by three freeze-pump-thaw cycles. Then, the solution of AIBN was added to the reaction mixture and the flask was immersed in an oil bath thermostated at 70 °C to start the reaction. The polymerization was quenched after a predetermined time (t = 21 h) by cooling and exposing the reaction mixture to air. The product was precipitated with cold diethyl ether and re-dissolved in chloroform and this cycle was repeated twice. The polymer was isolated, filtered, and then dried under vacuum to a constant mass.

2. *Synthetic pathway of PVP with star topology (starPVP)*

Thermally-initiated RAFT polymerization of VP using previously synthesized 1,3,5-benzyl tri(diethyldithiocarbamate), CTA2 (please see the synthetic pathway in our previous papers[1,2]) as a chain transfer agent, and AIBN as an initiator with molar ratios $[VP]_0/[CTA2]_0/[AIBN]_0 = 1200/5/1$) was carried out as follows. Before polymerization, VP was passed through an alumina column to remove the inhibitor. CTA2, VP, and dichloromethane (DCM) were placed in a Schlenk flask with a magnetic stirring bar. The solution was purged under nitrogen and purified by three freeze-pump-thaw cycles. Then, the solution of AIBN was added to the reaction mixture and the flask was immersed in an oil bath thermostated at 70 °C to start the reaction. The polymerization was quenched after a predetermined time (t = 10 h) by cooling and exposing the reaction mixture to air. The product was precipitated with cold diethyl ether and re-dissolved in chloroform, and this cycle was repeated twice. The polymer was isolated, filtered, and then dried under vacuum to a constant mass.

3. *Size Exclusion Chromatography (SEC)*

Molecular weights (M_w) and dispersities (*Đ*) of *lin*PVP and *star*PVP homopolymers were determined by size exclusion chromatography (SEC). Viscotek GPC Max VE 2001 and a Viscotek TDA 305 triple detection system (refractometer, viscosimeter, and low angle laser light scattering) were used for data collection, and OmniSec 5.12 for data processing. Two

T6000M general mixed columns were applied for separation. The measurements were carried out in DMF/LiBr (0.01M) as an eluent at 323 K with a flow rate of 0.5 mL/min.

4. Nuclear Magnetic Resonance (NMR)

Nuclear magnetic resonance (^{1}H NMR) spectra were collected using a Bruker Ascend 600 MHz spectrometer for the samples in $CDCl_3$ as a solvent with TMS internal standard. Standard experimental conditions and the standard Bruker program were used.

TGA data

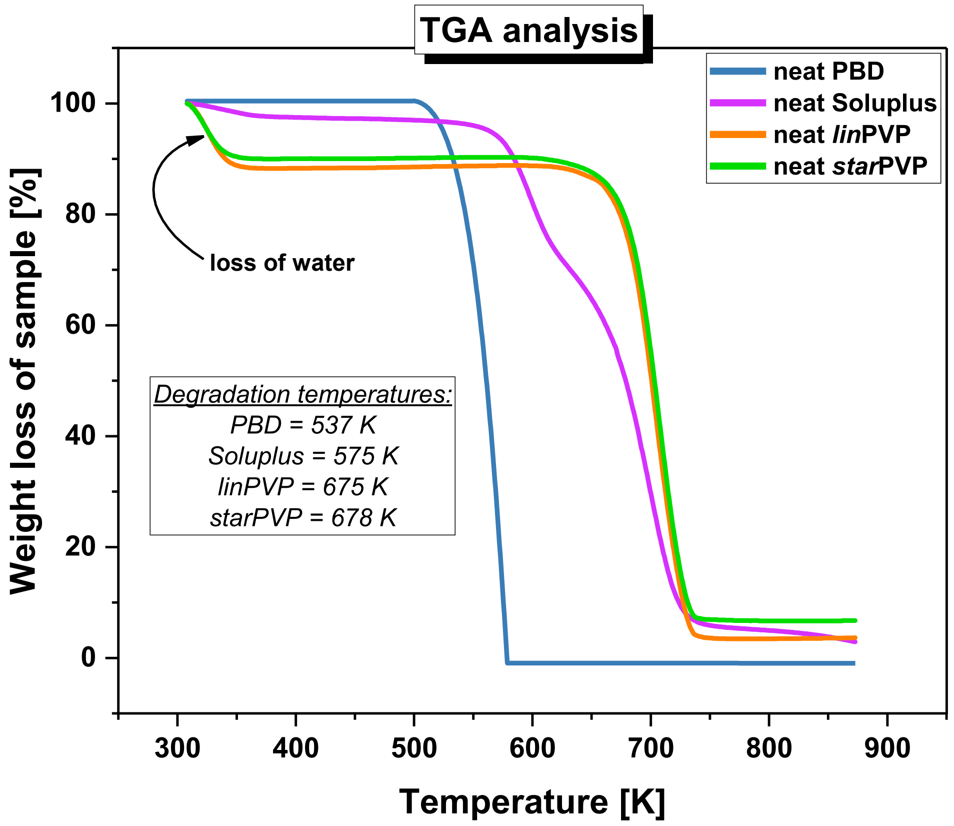

Figure S1. TGA traces of neat API and polymers (Soluplus, *lin*PVP, *star*PVP). The figure also presents the precise degradation temperatures of the individual compounds.

NMR data

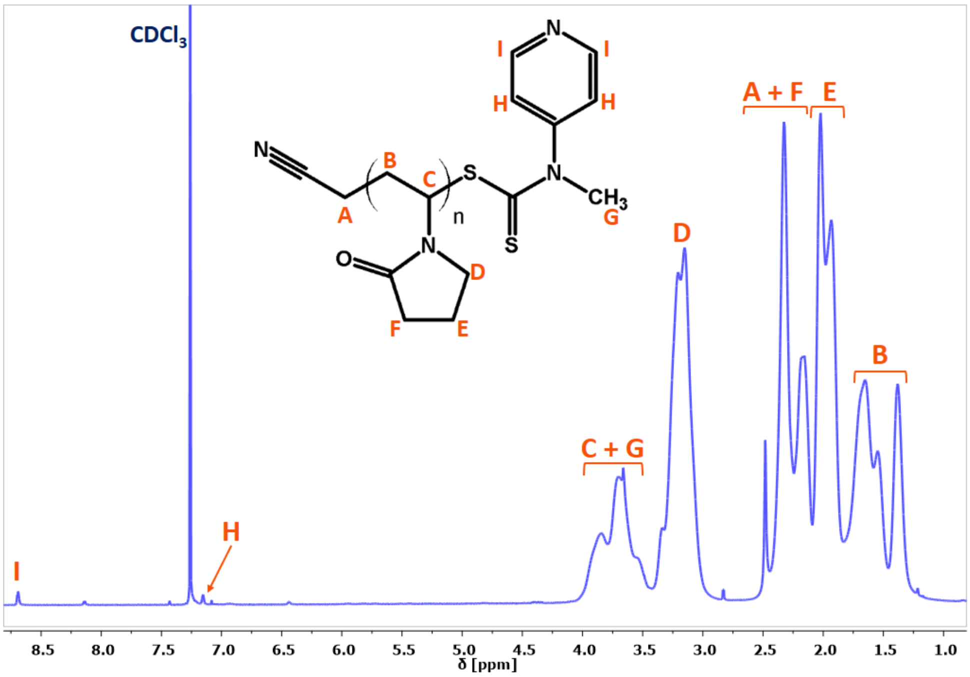

Figure S2. ^{1}H NMR spectrum of *lin*PVP in $CDCl_3$ (600 MHz).

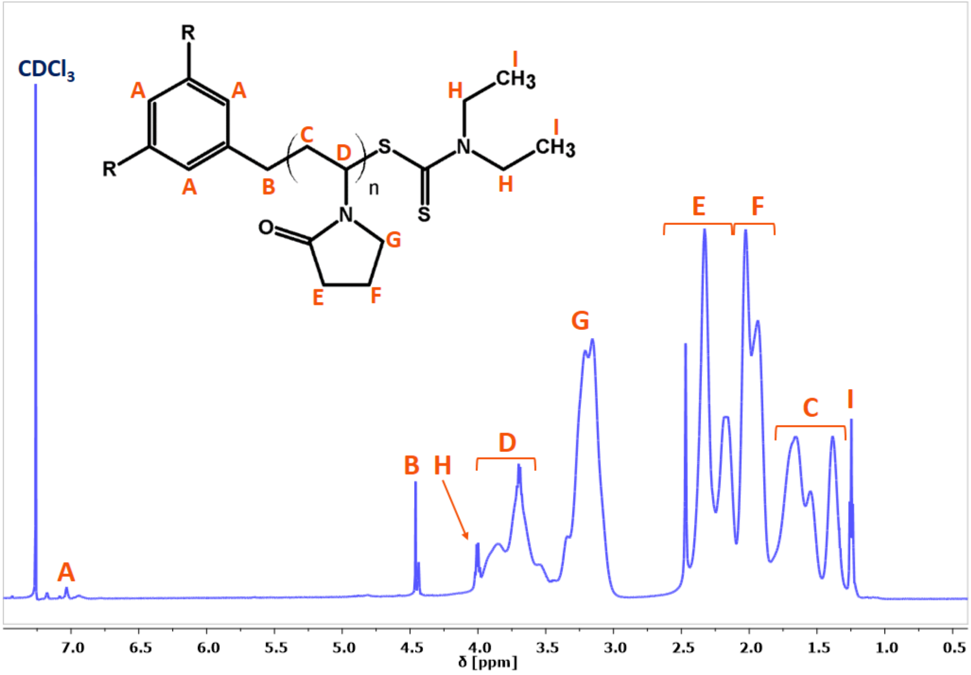

Figure S3. ^{1}H NMR spectrum of *star*PVP in $CDCl_3$ (600 MHz).

The ^{1}H NMR spectra confirm the structure of the synthesized macromolecules, i.e., *lin*PVP (**Figure S2**) and *star*PVP (**Figure S3**). In both figures, the chemical structures of the polymers are presented, and each signal is assigned to the corresponding proton group. Moreover, it is worth noting that a large amount of diethyl ether was used to purify the polymer

matrices after the polymerization process. However, as shown in **Figures S2** and **S3**, no signal is observed at the chemical shift of 1.21 ppm (which corresponds to the protons of diethyl ether),[3] indicating effective drying of the polymers after purification and the absence of residual solvents. Furthermore, **Figure S6** below shows the thermogram of a binary mixture with a high polymer content (40 wt%), where only the glass transition of the binary mixture is observed (T_g = 288 K), with no signs of solvent evaporation processes. A similar scenario was observed during the calorimetric measurements of the pure polymers (**Figure S9**), where a single, clearly visible signal corresponding to the glass transition of the polymers was detected, with no phase transitions that could indicate the presence of residual solvents. Thus, the calorimetric studies further confirm the purity of the obtained macromolecular compounds.

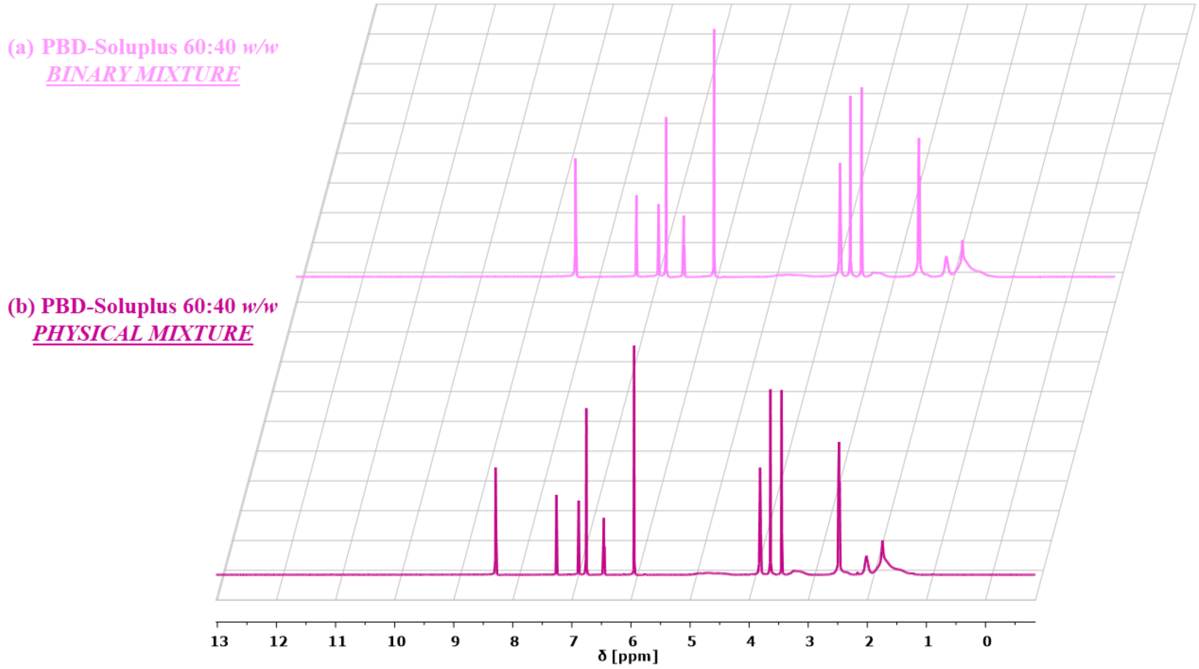

Figure S4. Comparison of ^{1}H NMR spectra of the PBD-Soluplus 60:40 *w/w* system: **(a)** binary and **(b)** physical mixtures.

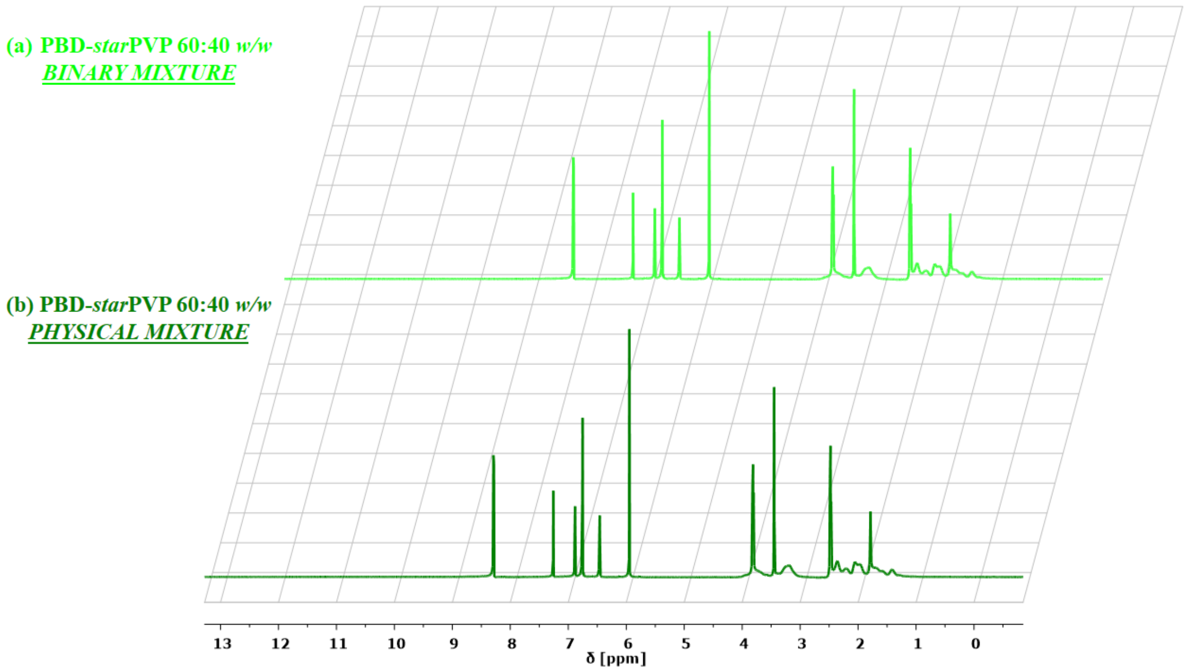

Figure S5. Comparison of ^{1}H NMR spectra of the PBD-*star*PVP 60:40 *w/w* system: **(a)** binary and **(b)** physical mixtures.

DSC data

Table S1. The calorimetric glass transition temperature values (heating rate, ϕ =10 K/min) for the examined PBD-polymer BMs in various weight ratios as well as neat polymers.

Binary mixture, *w/w*	**Glass transition temperature, T_g [K]**		
	PBD-Soluplus	PBD-*lin*PVP	PBD-*star*PVP
90:10	261	262	262
80:20	264	267	267
70:30	265	278	273
60:40	273	292	285
neat polymers	348	451	450

Table S2. The crystallization temperature values for neat PBD and PBD-polymer 90:10 and 80:20 *w/w* mixtures depending on the ϕ.

**PBD-EXCs 90:10 and 80:20 *w/w* mixtures**				
ϕ [K/min]	T_c [K]			
	neat PBD	PBD-Soluplus 90:10 *w/w*	PBD-Soluplus 80:20 *w/w*	PBD-*lin*PVP 90:10 *w/w*
2	313	345	348	336
4	317	347	349	345
6	–	349	350	–
8	326	351	351	–
20	338	–	–	–

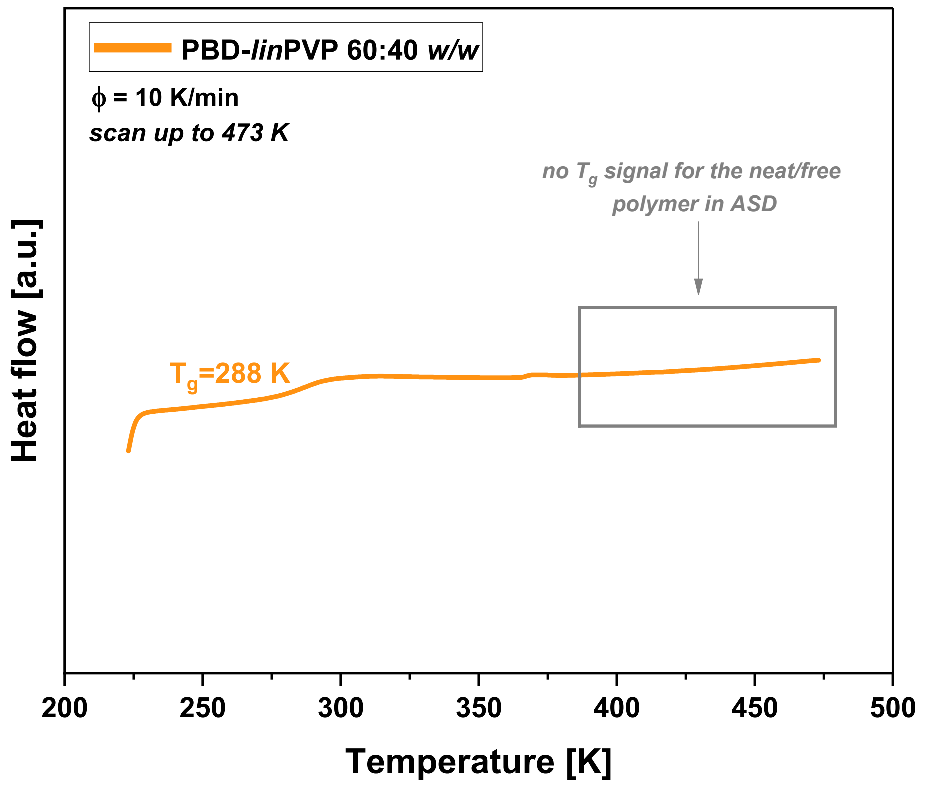

Figure S6. DSC thermogram (ϕ = 10 K/min) for PBD-*lin*PVP 60:40 *w/w* BM heated up to 473 K.

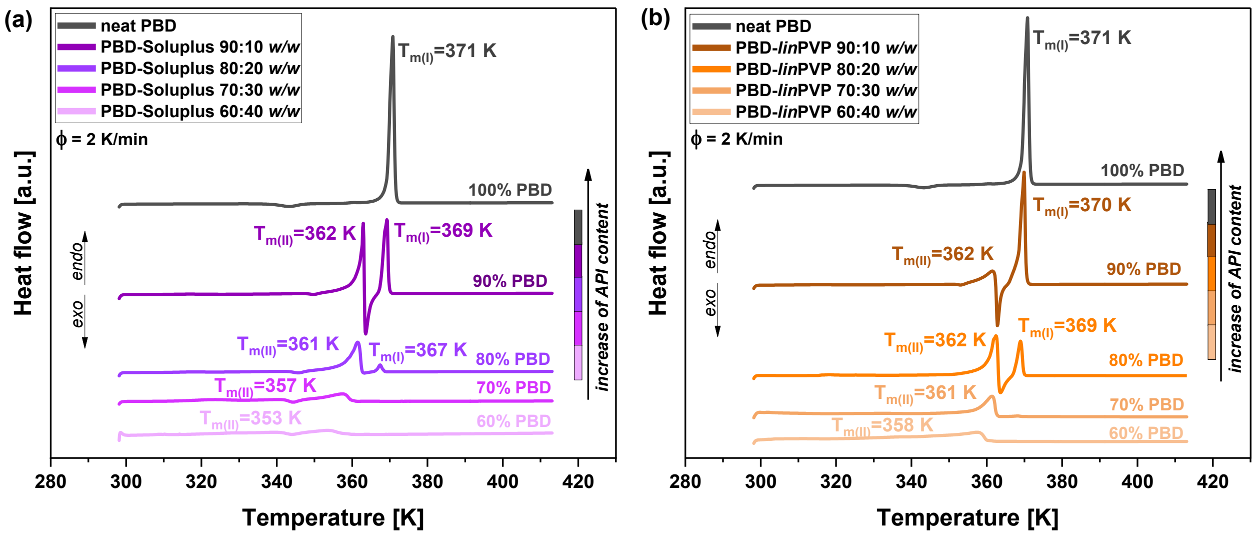

Figure S7. DSC thermograms of binary mixtures: **(a)** PBD-Soluplus and **(b)** PBD-*lin*PVP at a heating rate of 2 K/min. The data for neat PBD are also shown.

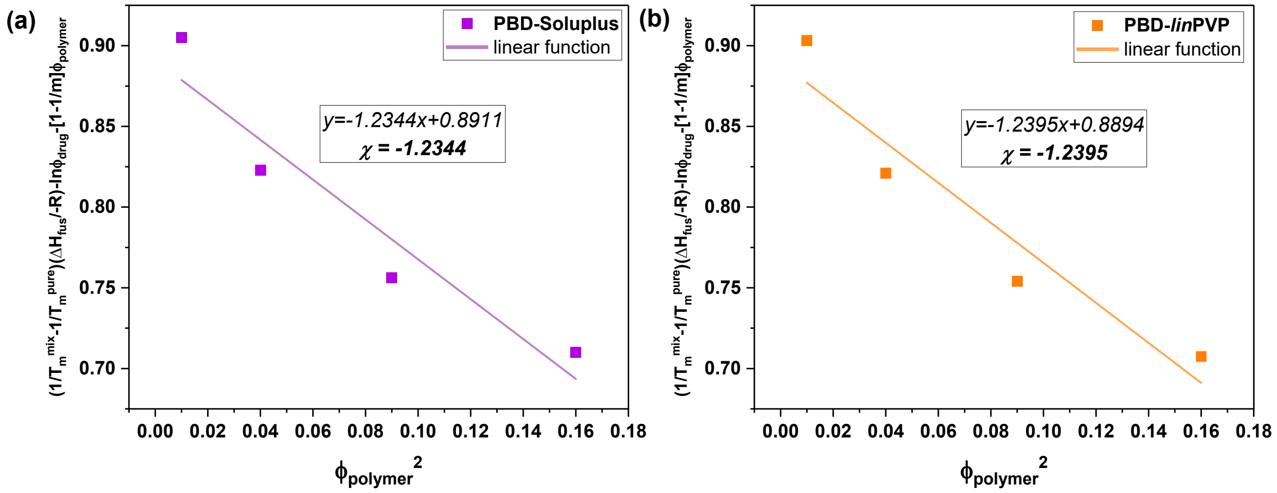

Figure S8. Graphs of $\left(\frac{1}{T_m^{mix}} - \frac{1}{T_m^{pure}}\right)\left(\frac{\Delta H_{fus}}{-R}\right) - ln\phi_{drug} - \left[1-\left(\frac{1}{m}\right)\right]\phi_{polymer}$ vs. $\phi_{polymer}^2$ to determine the χ values for **(a)** PBD-Soluplus and **(b)** PBD-*lin*PVP systems.

To confirm the miscibility of PBD with various types of polymers (Soluplus and PVP), the Flory–Huggins (F-H) theory was applied. This theory assumes that each molecule occupies a single site in the lattice and that the segments are randomly distributed. It is widely used to investigate the miscibility of a polymer with a solvent or another polymer by considering the change in Gibbs free energy before and after mixing.[4] However, if the solvent is replaced with another small molecule, such as a drug molecule, the F-H theory can also be employed to describe the thermodynamics of drug–polymer systems.[5]

The miscibility assessment based on the F-H theory can be carried out using a relatively simple method – namely, the melting point depression approach. It has been proposed that in a well-miscible drug–polymer system, the chemical potential of the drug in the presence of a polymeric carrier should be lower compared to its pure crystalline form, which results in a reduction of both the melting temperature and the enthalpy of fusion. Conversely, in an immiscible system, the chemical potential of the drug tends to remain constant, leading to negligible melting point depression. Based on this rationale, the change in Gibbs free energy can be expressed by the following equation:[6]

$$\Delta G = \mu_{liq} - \mu_{solid} = RTln(a) \tag{S1}$$

where μ_{liq} and μ_{solid} are the chemical potentials of molten drug and solid crystalline drug, respectively, and a refers to the activity coefficient of the species, which can be defined as:[6]

$$\ln(a) = -\frac{\Delta H_{fus}}{R}\left(\frac{1}{T_m^{mix}} - \frac{1}{T_m^{pure}}\right) \quad \text{(S2)}$$

where T_m^{mix} is melting temperature of drug in the drug-polymer mixture, T_m^{pure} is the melting point of pure crystalline drug, and ΔH_{fus} is the heat of fusion of the drug. In turn, the calculation of the F–H interaction parameter (χ) can be expressed by the following equation:[6]

$$\left(\frac{1}{T_m^{mix}} - \frac{1}{T_m^{pure}}\right) = \frac{-R}{\Delta H_{fus}}\left[ln\phi_{drug} + \left(1 - \frac{1}{m}\right)\phi_{polymer} + \chi\phi_{polymer}^2\right] \quad \text{(S3)}$$

where ϕ_{drug} and $\phi_{polymer}$ are the volume of the drug and polymer, respectively, and m is the ratio of the volume of the polymer to that of the drug. The value of χ can be determined from the slope of the graph by plotting $\left(\frac{1}{T_m^{mix}} - \frac{1}{T_m^{pure}}\right)\left(\frac{\Delta H_{fus}}{-R}\right) - ln\phi_{drug} - \left[1 - \left(\frac{1}{m}\right)\right]\phi_{polymer}$ versus $\phi_{polymer}^2$.

The melting point depression method has often been used as a means of evaluating the miscibility of dispersion systems, in which miscible/homogenous mixtures exhibit significant melting point depression with varying polymer concentrations, whereas in immiscible drug–polymer systems, the depression would be negligible due to the endothermic nature of mixing. Accordingly, the obtained χ value (near the drug's melting point) may reflect miscibility of a given system, where a positive value indicates immiscibility, while a negative χ suggests miscibility.[7]

Accordingly, to estimate the miscibility of the studied PBD–polymer systems, DSC measurements were carried out for the PBD–Soluplus and PBD–*lin*PVP binary mixtures at 90:10, 80:20, 70:30, and 60:40 *w/w*, using a heating rate of 2 K/min. The results of the thermal analysis are presented in **Figure S7**. As observed, the melting point of the drug gradually decreases with increasing polymer content, which is a primary indication of system miscibility. Furthermore, the obtained melting point data were used to plot $\left(\frac{1}{T_m^{mix}} - \frac{1}{T_m^{pure}}\right)\left(\frac{\Delta H_{fus}}{-R}\right) - ln\phi_{drug} - \left[1 - \left(\frac{1}{m}\right)\right]\phi_{polymer}$ versus $\phi_{polymer}^2$ dependencies, with the resulting plots shown in **Figure S8**. As previously mentioned, the interaction parameters were determined from the slope values and were found to be −1.2344 and −1.2395 for the PBD–Soluplus and PBD–*lin*PVP systems, respectively. Thus, according to the F–H theory, the obtained negative χ values suggest that both the commercial copolymer Soluplus and the synthesized PVPs exhibit good miscibility with the studied active pharmaceutical ingredient – PBD.

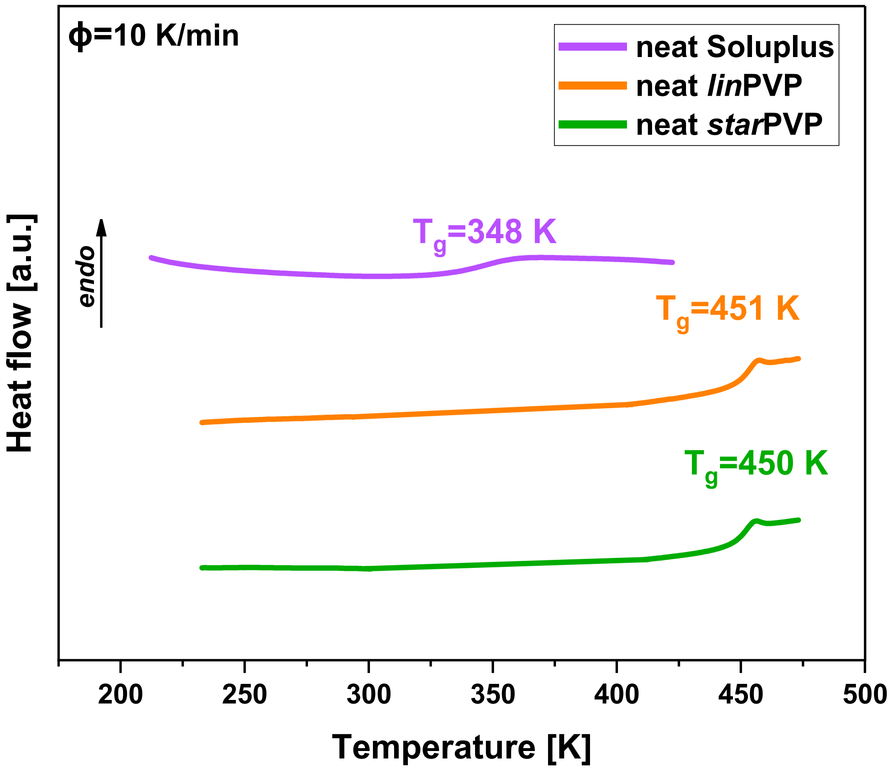

Figure S9. DSC thermograms (ϕ = 10 K/min) collected for neat polymers.

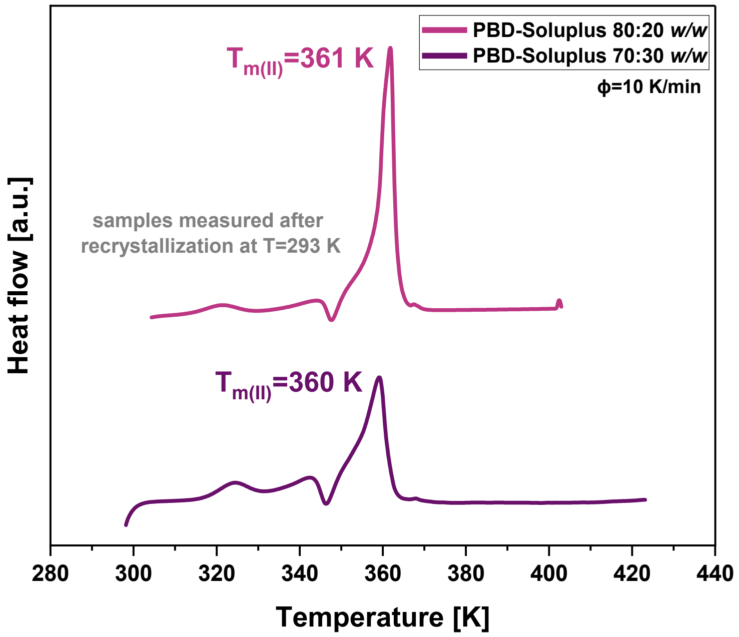

Figure S10. DSC thermograms (ϕ = 10 K/min) of PBD-Soluplus BMs (80:20 and 70:30 *w/w*) after recrystallization at room temperature.

FTIR data

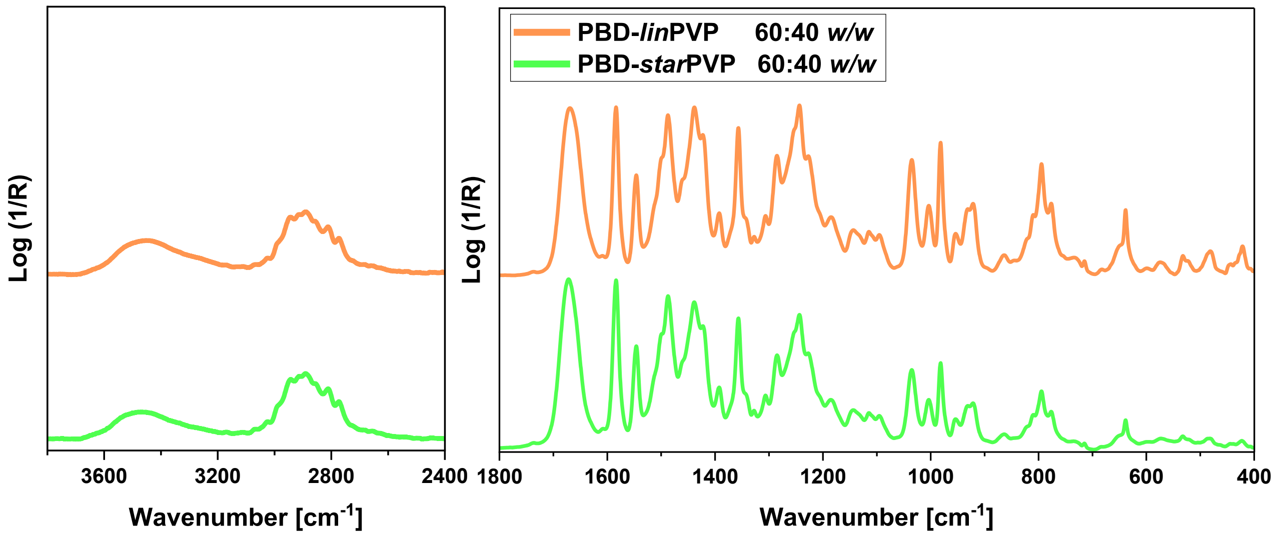

Figure S11. FTIR spectra of PBD-*lin*PVP and PBD-*star*PVP 60:40 *w/w* BMs, presented in the ranges of (left) 3800–2400 cm^{-1} and (right) 1800–400 cm^{-1}.

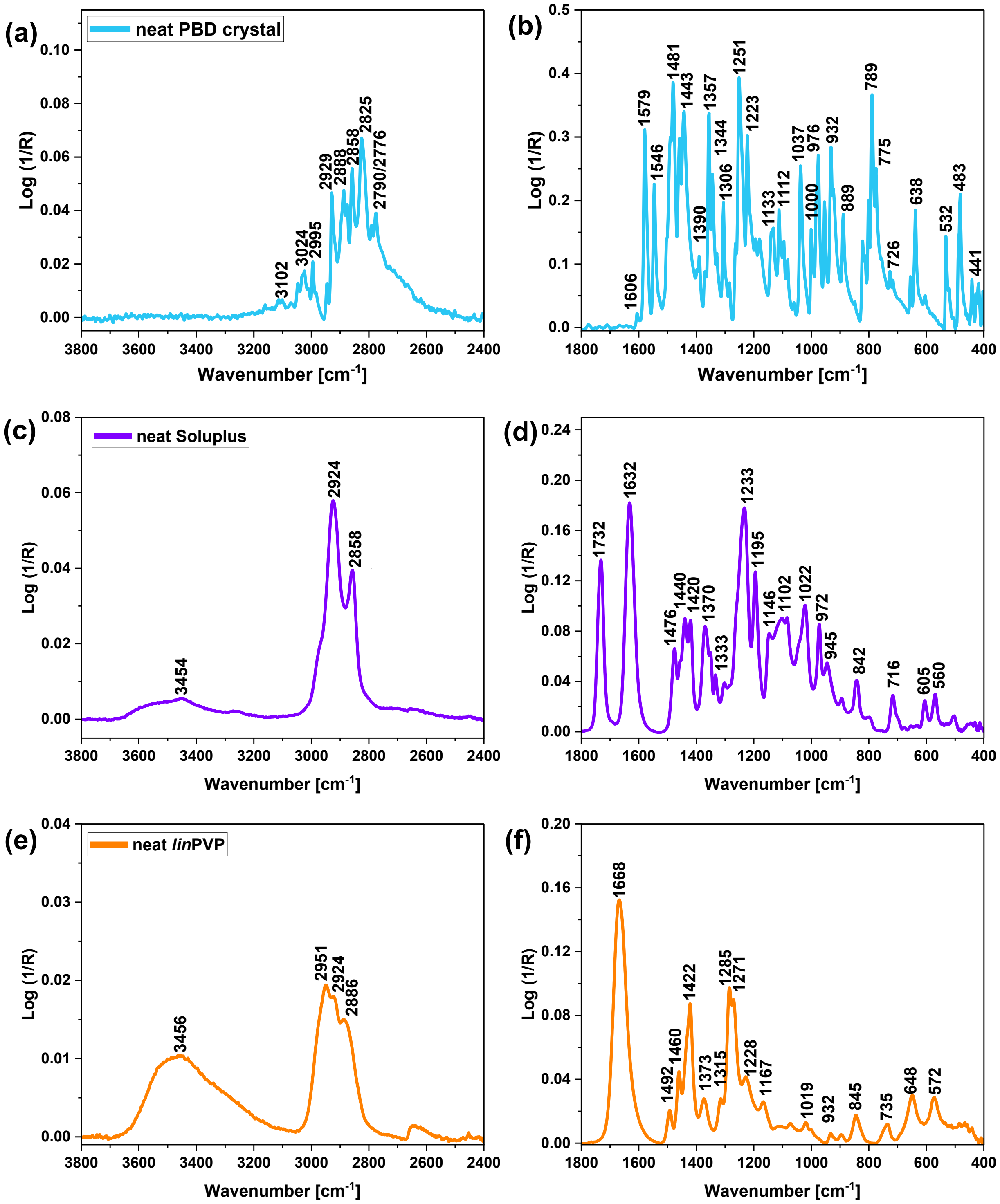

Figure S12. FTIR spectra of crystalline PBD as well as Soluplus and *lin*PVP samples. Data were presented in two spectral regions: **(a,c,e)** 3800–2400 cm^{-1} and **(b,d,f)** 1800–400 cm^{-1}.

The FTIR spectrum of neat crystalline PBD shows prominent absorption signals at several wavenumbers: 3102 and 3024 cm^{-1} (aromatic C-H stretching), 2995 cm^{-1} (asymmetric and symmetric CH_2 stretching), 2929, 2888 and 2858 cm^{-1} (C-H stretching), 2825, 2790 and 2776 cm^{-1} (C-H stretching of 1,3-dioxolane ring, i.e., C-H near oxygens), 1579 cm^{-1} (C=C and C=N ring stretching), 1546 cm^{-1} (conjugated C=C stretching), 1481 cm^{-1} (C-N stretching), 1357 cm^{-1} (additional conjugated C=C stretching), 1306 cm^{-1} (C-O stretching), 1251 cm^{-1} (asymmetric C-O stretching), 1112 cm^{-1} (C-N-C stretching), 1037 cm^{-1} (aromatic C-H stretching), and 976-483 cm^{-1} (C=C and C=N stretching, aromatic out-of-plane C-H deformation) (**Figure S12a,b**).[8,9]

The FTIR spectrum of Soluplus displays the stretching bands at 3454 cm^{-1} (intermolecular H-bonded O-H groups) as well as at 2924 and 2858 cm^{-1} (the asymmetric and symmetric C-H stretching) (**Figure S12c,d**). The strong absorption peaks in this polymer reflect the carbonyl stretching vibrations of the caprolactam ring (C(=O)N tertiary amide group, 1632 cm^{-1}) and the ester group ($OC(=O)CH_3$, 1732 cm^{-1}). The C-O-C stretching can be visible at 1476 cm^{-1}, and the C-H bending is detected at 1440 cm^{-1}. The ester C–O stretching vibrations are observed at 1233 and 1102 cm^{-1}.[9–13] In turn, PVP with linear topology is characterized by the IR peaks at 3456 cm^{-1} (O-H stretching of residual water), 2951 cm^{-1} (asymmetric CH_2 stretching of pyrrolidone ring), 2924 cm^{-1} (symmetric CH_2 stretching of chain), and 2886 cm^{-1} (C-H stretching), 1668 cm^{-1} (C=O stretching), 1492, 1460, 1422 cm^{-1} (C-N stretching and CH_2 scissoring), 1373 cm^{-1} (C-H bending), 1285, 1271, 1228 and 1167 cm^{-1} (C-N stretching and CH_2 wagging), 1019 cm^{-1} (C-C stretching and CH_2 rocking), 932 cm^{-1} (C-C ring breathing), 845 cm^{-1} (C-C ring stretching), 735 cm^{-1} (C-C chain stretching), 648 and 572 cm^{-1} (N-C=O bending, ring deformation) (**Figure S12e,f**).[9,14,15]

BDS data

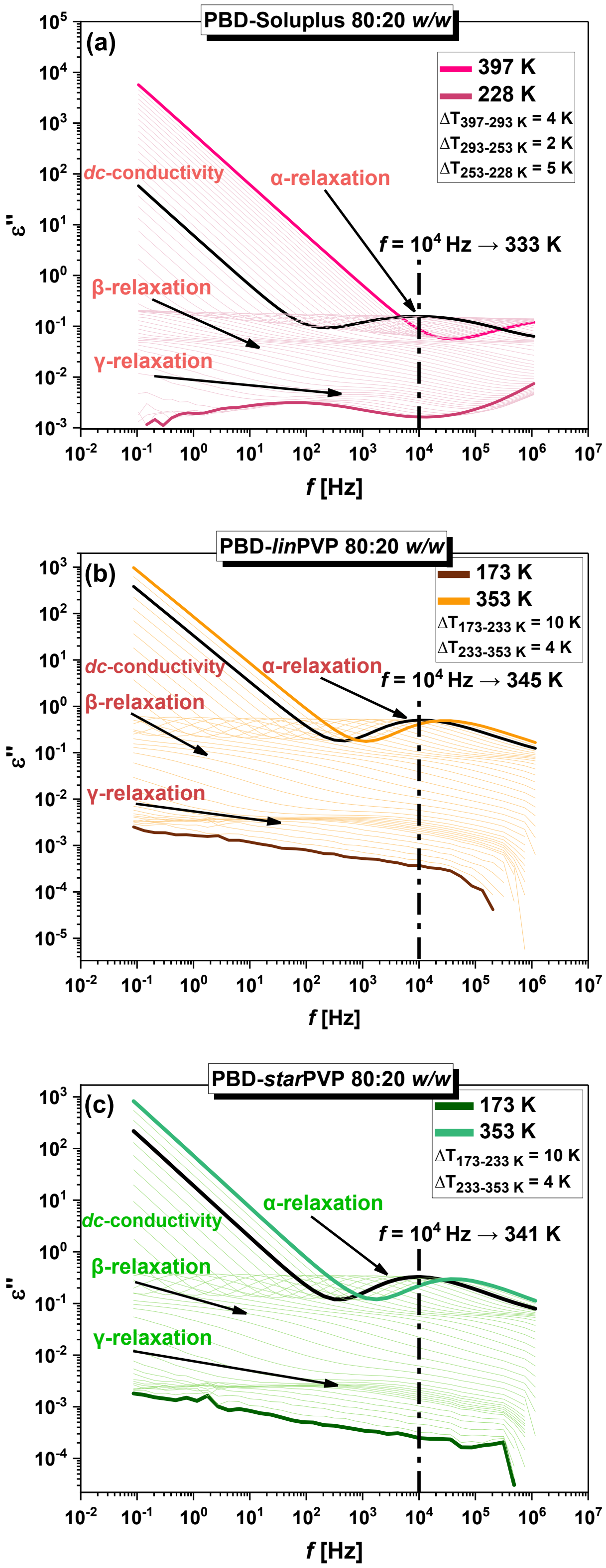

Figure S13. Dielectric loss spectra of **(a)** PBD-Soluplus **(b)**, PBD-*lin*PVP, **(c)**, and PBD-*star*PVP 80:20 *w/w* BMs.

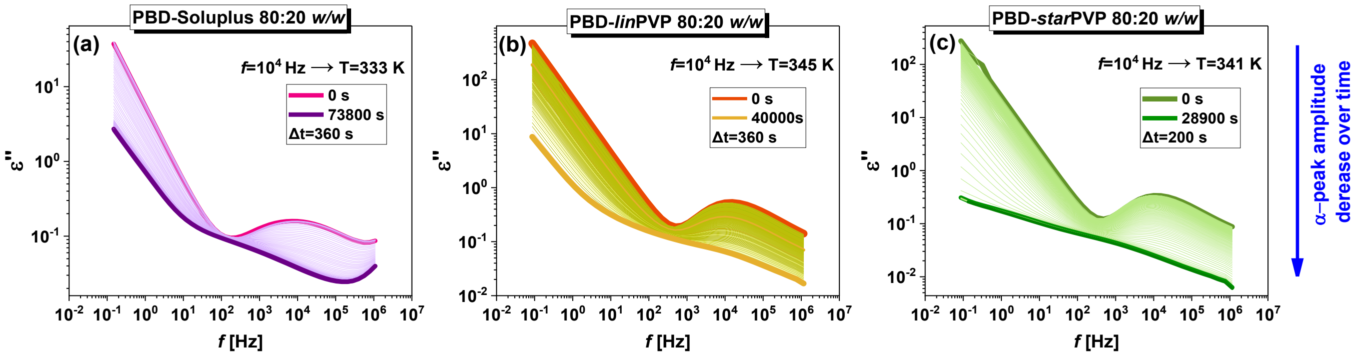

Figure S14. Time evolution of the imaginary (ε'') part of the complex dielectric permittivity plotted versus frequency during the isothermal crystallization of **(a)** PBD-Soluplus 80:20 *w/w*, **(b)** PBD-*lin*PVP 80:20 *w/w*, **(c)** PBD-*star*PVP 80:20 *w/w* at indicated temperatures (f=10^4 Hz).

Table S3. Summary of $T_{c(BDS)}$, $T_{g(DSC)}$ (ϕ = 10 K/min) and $\frac{T_{c(BDS)}}{T_{g(DSC)}}$ values for individual 90:10 and 80:20 *w/w* binary systems.

	**API-polymer weight ratio**	$T_{c\,(BDS)}$ **[K]**	$T_{g\,(DSC)}$ **[K]**	$\frac{T_{c\,(BDS)}}{T_{g\,(DSC)}}$
**PBD-Soluplus**	90:10	328	261	1.26
	80:20	333	264	1.26
**PBD-*lin*PVP**	90:10	333	262	1.27
	80:20	345	267	1.29
**PBD-*star*PVP**	90:10	333	262	1.27
	80:20	341	267	1.28

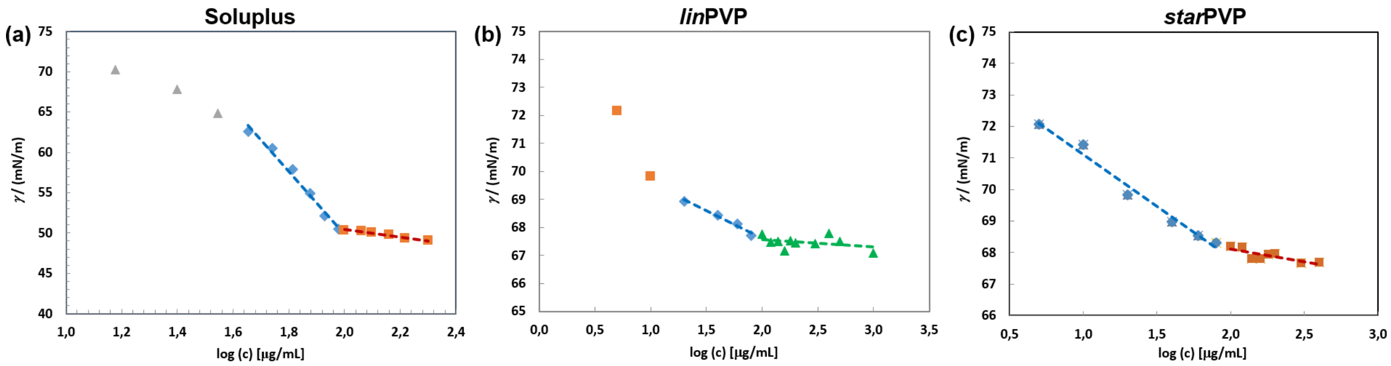

Figure S15. Determination of CMC by measuring the surface tension (γ) at 25 °C of serial dilutions of **(a)** Soluplus, **(b)** *lin*PVP, and **(c)** *star*PVP. The dashed lines are the best linear fits in the two concentration ranges.

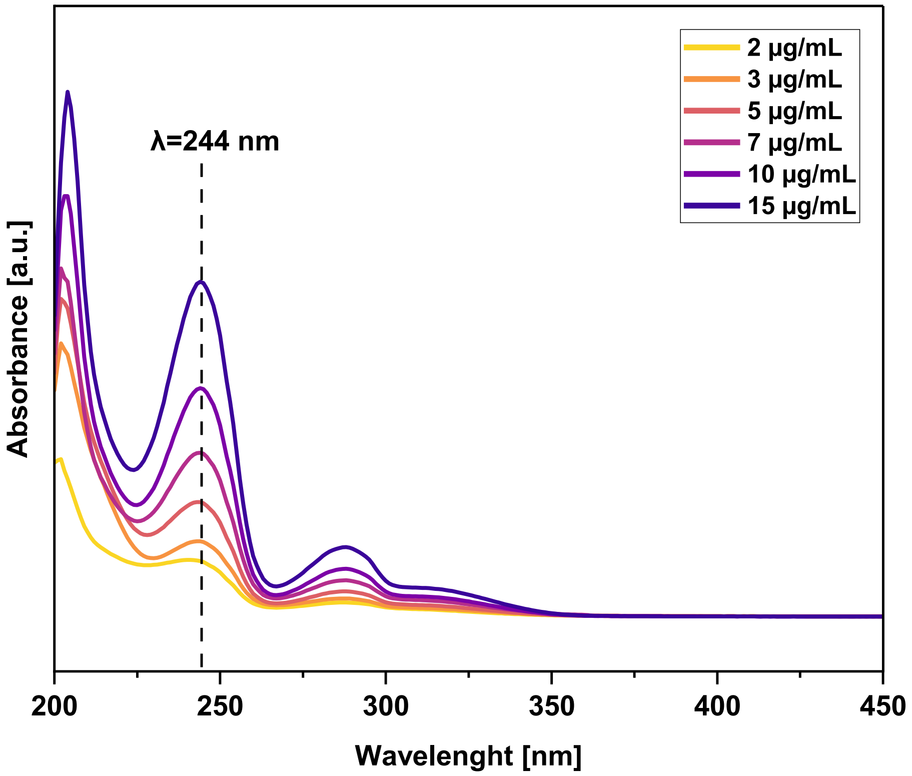

Figure S16. UV-Vis spectra for PBD at different concentrations (in the range of 2–15 µg/mL; solutions in ethanol solvent).

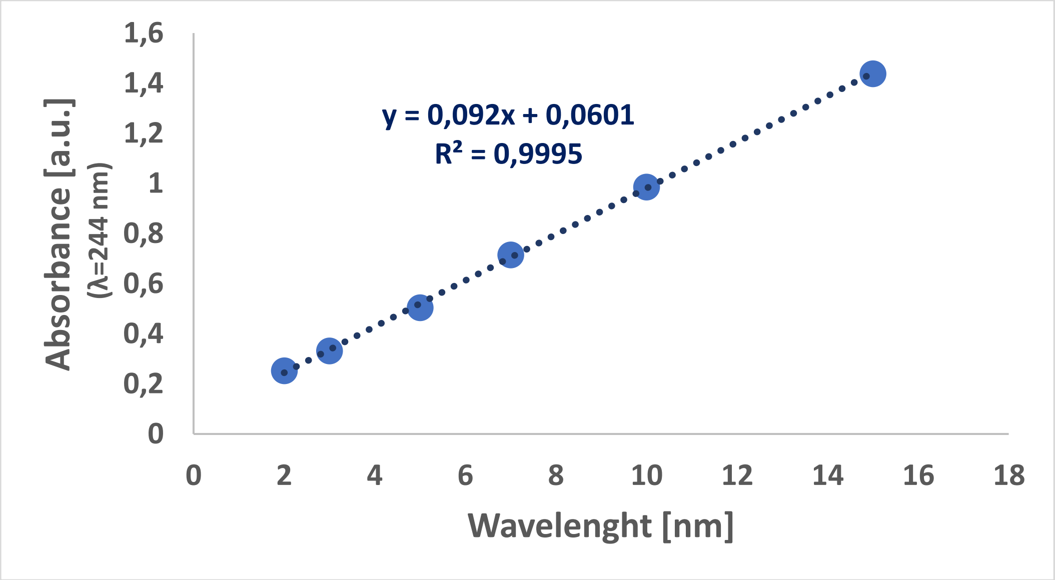

Figure S17. The calibration curve for PBD in ethanol.

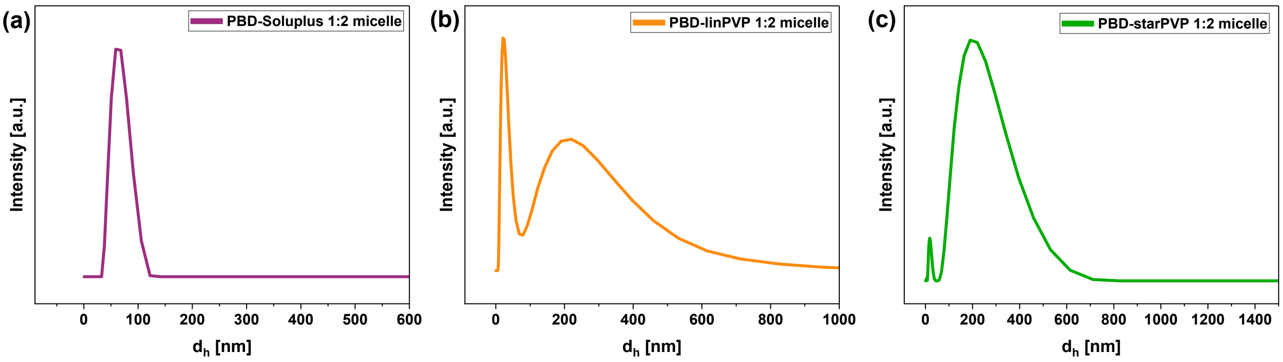

Figure S18. Hydrodynamic diameters (d_h) of obtained micellar systems: **(a)** PBD-Soluplus 1:2, **(b)** PBD-*lin*PVP 1:2, **(c)** PBD-*star*PVP 1:2 (aqueous solutions with a concentration of $c = 1$ mg/mL).

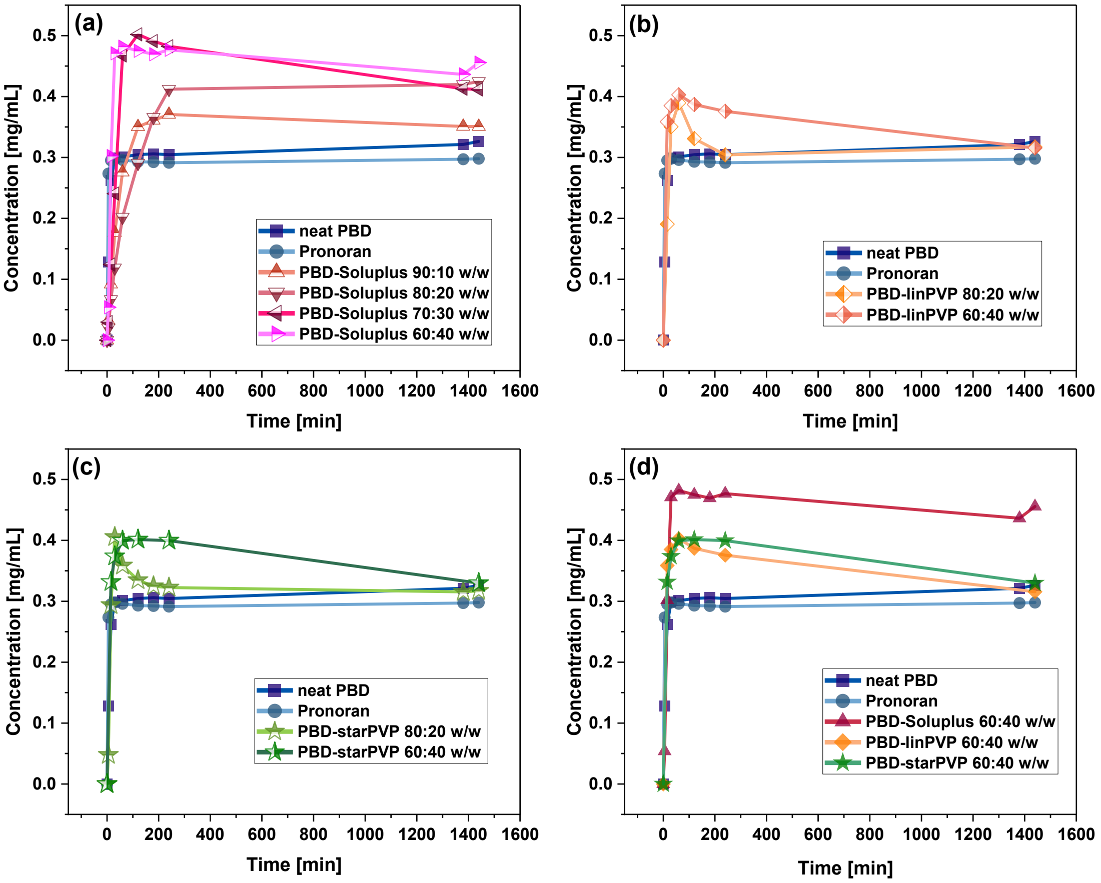

Figure S19. Drug release profile (drug concentration vs. time) from various polymer matrices in **(a)** PBD-Soluplus; **(b)** PBD-*lin*PVP; and **(c)** PBD-*star*PVP BMs. Panel **(d)** presents a comparison of drug release profiles from various polymer matrices for 60:40 *w/w* BMs. Each panel contains two reference samples: neat crystalline PBD and the API Pronoran.

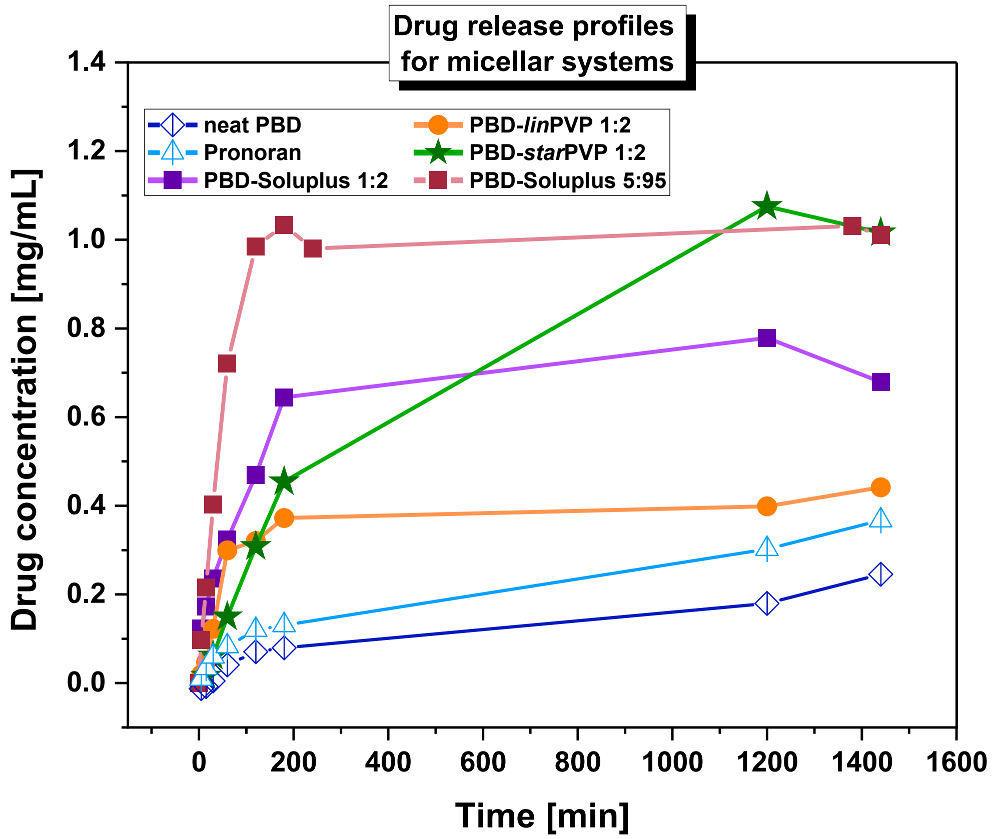

Figure S20. Drug release profiles (drug concentration vs. time) for micellar systems with various polymer matrices, as well as for the reference samples: neat PBD and the Pronoran tablet.

DSC data (drug release studies)

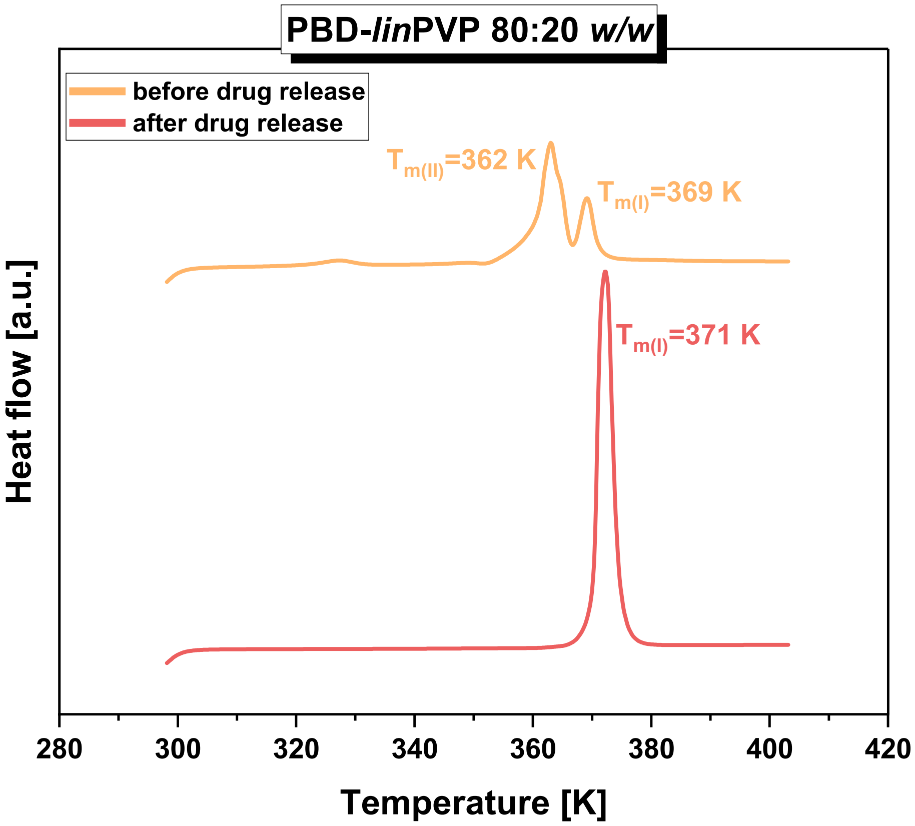

Figure S21. DSC data (ϕ = 10 K/min) for the PBD–*lin*PVP 80:20 *w/w* binary mixture before (orange line) and after (red line) the release process.

References:

(1) Orszulak, L.; Lamrani, T.; Tarnacka, M.; Hachuła, B.; Jurkiewicz, K.; Zioła, P.; Mrozek-Wilczkiewicz, A.; Kamińska, E.; Kamiński, K. The Impact of Various Poly(Vinylpyrrolidone) Polymers on the Crystallization Process of Metronidazole. *Pharmaceutics* **2024**, *16* (1). https://doi.org/10.3390/pharmaceutics16010136.

(2) Orszulak, L.; Lamrani, T.; Bernat, R.; Tarnacka, M.; Żakowiecki, D.; Jurkiewicz, K.; Zioła, P.; Mrozek-Wilczkiewicz, A.; Zięba, A.; Kamiński, K.; Kamińska, E. The Influence of PVP Polymer Topology on the Liquid Crystalline Order of Itraconazole in Binary Systems. *Mol. Pharm.* **2024**, *21* (6), 3027–3039. https://doi.org/10.1021/acs.molpharmaceut.4c00215.

(3) Gottlieb, H. E.; Kotlyar, V.; Nudelman, A. NMR Chemical Shifts of Common Laboratory Solvents as Trace Impurities. *J. Org. Chem.* **1997**, *62* (21), 7512–7515. https://doi.org/10.1021/jo971176v.

(4) Sarode, A. L.; Sandhu, H.; Shah, N.; Malick, W.; Zia, H. Hot Melt Extrusion (HME) for Amorphous Solid Dispersions: Predictive Tools for Processing and Impact of Drug-Polymer Interactions on Supersaturation. *Eur. J. Pharm. Sci.* **2013**, *48* (3), 371–384. https://doi.org/10.1016/j.ejps.2012.12.012.

(5) Zhao, Y.; Inbar, P.; Chokshi, H. P.; Malick, A. W.; Choi, D. S. Prediction of the Thermal Phase Diagram of Amorphous Solid Dispersions by Flory-Huggins Theory. *J. Pharm. Sci.* **2011**, *100* (8), 3196–3207. https://doi.org/10.1002/jps.22541.

(6) Meng, F.; Dave, V.; Chauhan, H. Qualitative and Quantitative Methods to Determine Miscibility in Amorphous Drug-Polymer Systems. *Eur. J. Pharm. Sci. Off. J. Eur. Fed. Pharm. Sci.* **2015**, *77*, 106–111. https://doi.org/10.1016/j.ejps.2015.05.018.

(7) Bansal, K.; Baghel, U. S.; Thakral, S. Construction and Validation of Binary Phase Diagram for Amorphous Solid Dispersion Using Flory-Huggins Theory. *AAPS PharmSciTech* **2016**, *17* (2), 318–327. https://doi.org/10.1208/s12249-015-0343-8.

(8) Bhargavi, C.; Raghuveer, P. Enhancing Nose-To-Brain Delivery of Piribedil: Development of a Nanosuspension Dispersed in Nasal in-Situ Gelling System. *Int. J. Appl. Pharm.* **2024**, *16* (3), 86–101. https://doi.org/10.22159/ijap.2024v16i3.50242.

(9) Socrates, G. Infrared and Raman Characteristic Group Frequencies : Tables and Charts; 2001.

(10) Desai, P.; Chatterjee, B. Comparison of Two Grafted Copolymers, Soluplus and Kollicoat IR, as Solid Dispersion Carriers of Arteether for Oral Delivery Prepared by Different Solvent-Based Methods. *ACS Omega* **2023**, *8* (48), 45337–45347. https://doi.org/10.1021/acsomega.3c04110.

(11) Thakral, N. K.; Ray, A. R.; Bar-Shalom, D.; Eriksson, A. H.; Majumdar, D. K. Soluplus-Solubilized Citrated Camptothecin - A Potential Drug Delivery Strategy in Colon Cancer. *AAPS PharmSciTech* **2012**, *13* (1), 59–66. https://doi.org/10.1208/s12249-011-9720-0.

(12) Lin, S. Y.; Lin, H. L.; Chi, Y. T.; Hung, R. Y.; Huang, Y. T.; Hsieh, W. H.; Kao, C. Y. Influence of Soluplus on Solid-State Properties and Physical Stability of Indomethacin-Saccharin Co-Crystal Formation Prepared by Air-Drying Process. *J. Pharm. Innov.* **2016**, *11* (2), 109–119. https://doi.org/10.1007/s12247-016-9243-z.

(13) Shamma, R. N.; Basha, M. Soluplus®: A Novel Polymeric Solubilizer for Optimization of Carvedilol Solid Dispersions: Formulation Design and Effect of Method of Preparation. *Powder Technol.* **2013**, *237*, 406–414. https://doi.org/https://doi.org/10.1016/j.powtec.2012.12.038.

(14) Rahma, A.; Munir, M. M.; Khairurrijal; Prasetyo, A.; Suendo, V.; Rachmawati, H. Intermolecular Interactions and the Release Pattern of Electrospun Curcumin-Polyvinyl(Pyrrolidone) Fiber. *Biol. Pharm. Bull.* **2016**, *39* (2), 163–173. https://doi.org/10.1248/bpb.b15-00391.

(15) Borodko, Y.; Habas, S. E.; Koebel, M.; Yang, P.; Frei, H.; Somorjai, G. A. Probing the Interaction of Poly (Vinylpyrrolidone) with Platinum Nanocrystals by \mbox{UV}-\mbox{R}aman and \mbox{FTIR}. *J. Phys. Chem. B* **2006**, *110*, 23052–23059.
